# The mmu_circRNA_37492/hsa_circ_0012138 function as potential ceRNA to attenuate obstructive renal fibrosis

**DOI:** 10.1038/s41419-022-04612-3

**Published:** 2022-03-04

**Authors:** Xu Cheng, Kai Ai, Lei Yi, Wentao Liu, Yijian Li, Yinhuai Wang, Dongshan Zhang

**Affiliations:** 1 Department of Emergency Medicine, The Second Xiangya Hospital, Central South University, Changsha, Hunan 410011 China; 2 Emergency Medicine and Difficult Diseases Institute, The Second Xiangya Hospital, Central South University, Changsha, Hunan 410011 China; 3 Department of Urology, The Second Xiangya Hospital, Central South University, Changsha, Hunan 410011 China

**Keywords:** End-stage renal disease, miRNAs

## Abstract

Circular RNAs (circRNAs) are involved in the pathogenesis of certain renal diseases, however, the function and mechanism of them in renal fibrosis remains largely unknown. In the present study, RNA expression data in unilateral ureteral obstruction (UUO) kidneys was obtained from our previous circRNA Microarray and public Gene Expression Omnibus datasets to construct a ceRNA network. The effects of target circRNA as long as the homologous human circRNA on renal fibrosis was examined in vitro and in vivo. Gene ontology (GO) and Kyoto Encyclopedia of Genes and Genomes (KEGG) enrichment analysis was further performed among genes regulated by the human circRNA. We found that circRNA_37492, showing well connection degree in the ceRNA network, was abundant expression and high sequence conservation. We observed that the expression of circRNA_37492 was induced by the TGF-β1 or UUO in BUMPT cells and C57BL/6 mice, respectively. In vitro, cytoplasmic circRNA_37492 inhibited type I, III collagen and fibronectin deposition by sponging miR-7682-3p and then upregulated its downstream target Fgb. In vivo, overexpression of circRNA_37492 attenuated fibrotic lesions in the kidneys of UUO mice via targeting miR-7682-3p/Fgb axis. Furthermore, hsa_circ_0012138, homologous with circRNA_37492, may potentially target miR-651-5p/FGB axis in human renal fibrosis. Not only that, GO and KEGG enrichment revealed that hsa_circ_0012138-regulated genes were previously demonstrated to related to the fibrosis. In conclusion, we for the first time demonstrated that circRNA_37492 attenuated renal fibrosis via targeting miR-7682-3p/Fgb axis, and the homologous hsa_circRNA_0012138 was speculated as a possible ceRNA to regulate multiple gene expressions and involve in human renal fibrosis, suggesting that circRNA_37492/hsa_circ_0012138 may serve as potent therapy target for obstructive renal fibrosis disease.

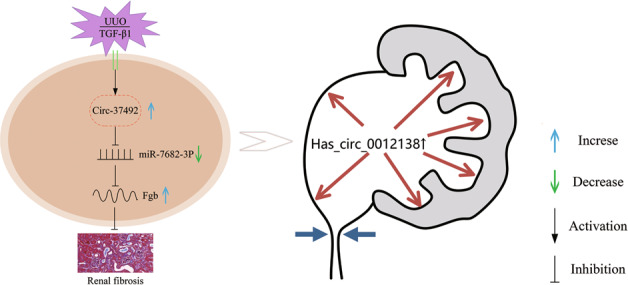

## Introduction

Chronic kidney disease (CKD) is a major public health problem with significant morbidity and mortality all over the world, obstructive nephropathy (ON) is the main cause of CKD [1]. Renal fibrosis is the terminal pathological stage of various CKDs [2]. Although previous literatures have reported that inflammation, oxidative stress, cytokine signal transduction, and fibroblast activation were important risk factor in the development of renal fibrosis [3–5], the mechanism of it is unclear.

Circular RNA (circRNA), lacking 5′cap and a 3′poly (A) tail, is a stable ring structure with highly conservative sequences across species [6, 7], and implicated in the pathophysiology of various renal diseases (e.g., lupus nephritis, diabetic nephropathy, and focal segmental glomerulus sclerosis) [8–15]. Our recent study reported that mmu_circRNA_30032 promoted unilateral ureteral obstruction (UUO)-mice kidney fibrogenesis via microRNA (miRNA) sponge [16], however, what role the circRNAs exactly played in renal fibrosis caused by mice-UUO and human ON remains largely unknown.

In this study, non-coding RNA expression profiles from public Gene Expression Omnibus datasets and our previous circRNA array data was used to comprehensively evaluate the landscape of competing endogenous RNAs (ceRNAs) network and to determine the key ceRNA pathway with targeted circRNA. After selection, the key circRNA, miRNA and mRNA still go through further experimental validation in vitro and vivo. In addition, to push these results towards clinical use, the homologous circRNA in human was searched out and verified by experiments and bioinformatics. Identification of these circRNAs and their role, may reveal novel therapeutic targets for renal fibrosis caused by ON.

## Results

### Construction of a circular RNA-associated ceRNA regulatory network

**Fig. 1:**
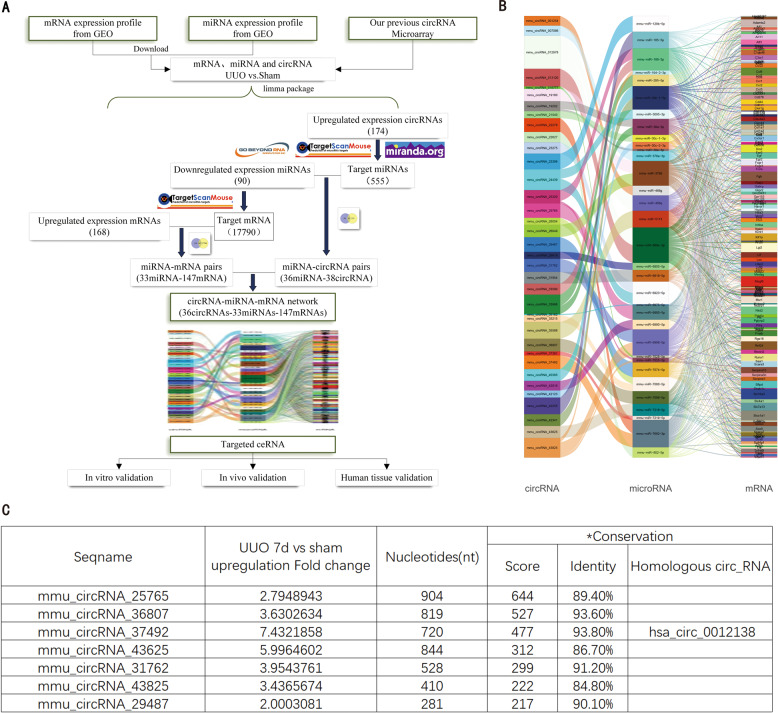
Construction of a circular RNA-associated ceRNA regulatory network in C57BL/6 mice UUO model and screening for targeted circular RNA. **A** Overview of the targeted circular RNA mining and further validation of its ceRNA regulatory pathway. **B** Sankey diagram for the ceRNA network in UUO model. Each rectangle represents a circular RNA, microRNA, and mRNA, and the line connecting them represents form of a regulatory pathway. The connection degree of each gene is displayed based on the size of the rectangle. **C** List of selected circular RNAs (fold change >2, nucleotides <1000) from sankey diagram and the top 7 circular RNAs ranked by sequence conservation between human and mouse species. *Conservation of sequence was tested by blast function in circBase website (http://circrna.org/cgi-bin/webBlat). In the blast results, the higher scores, the more deemed homologous hsa_circ_RNA when identity >80%.

As the flow diagram (Fig. 1A) shows, RNA expression data in UUO kidneys and control samples were obtained from circRNA Microarray and public Gene Expression Omnibus datasets. By differential expression analysis, we found that 174 circRNAs were upregulated (Fig. S1A). The predicted miRNAs with binding sites were figured out to 555, while 90 miRNAs were actually downregulated in GSE42716 and GSE162794 and 36 miRNAs left when taking intersection of them. The 90 miRNAs were predicted to target 17790 mRNAs, however, only 168 mRNA was upregulated and 147 of them bind to miRNAs. Next, the candidate 38 circRNAs-36 miRNAs were intersected with candidate 33 miRNAs-147 mRNAs and the 36 circRNA-33 miRNA-147 mRNA ceRNA network was established (Fig. 1B).

These circular RNAs (fold change >2, nucleotides <1000) from sankey diagram were selected and the top 7 circular RNAs ranked by sequence conservation score were shown in Fig. 1C. Among them, circRNA_37492 with a homologous hsa_circ_0012138 was the most potential target, considering the fold change and conservation score. The top 10 miRNAs and mRNAs ranked by connection degree in ceRNA network were determined for further in vitro validation. In addition, to assess the stability of network, we selected several nodes for correlation analysis, which showed that circRNA_37492 was positively correlated with different targeted genes (Fgb & Megf6), and Fgb was positively correlated with different circRNAs (circRNA_22278 & circRNA_37492), suggesting the ceRNA network of better stability (Fig. S1B–D).

### The circRNA_37492 was induced in TGF-β1-treated BUMPT cells and UUO mice

The fluorescent in situ hybridization (FISH) and confocal microscopy showed that circRNA_37492 was mainly enriched in the cytoplasm of Boston University mouse proximal tubular (BUMPT) cells (Fig. S2A). In addition, following stimulation with external TGF-β1 for different times (0, 6, 12, and 24 h), or UUO at different days (0, 3d, 7d), circRNA_37492 expression was induced and detected by real-time qPCR (RT-qPCR) in BUMPT cells and C57BL/6 mice, respectively (Fig. S2B, S2C). These data suggested a role that circRNA_37492 may play in the development of the obstruction-induced fibrotic kidney.

### Opposite fibrotic effects in circRNA_37492 inhibition & overexpression

**Fig. 2:**
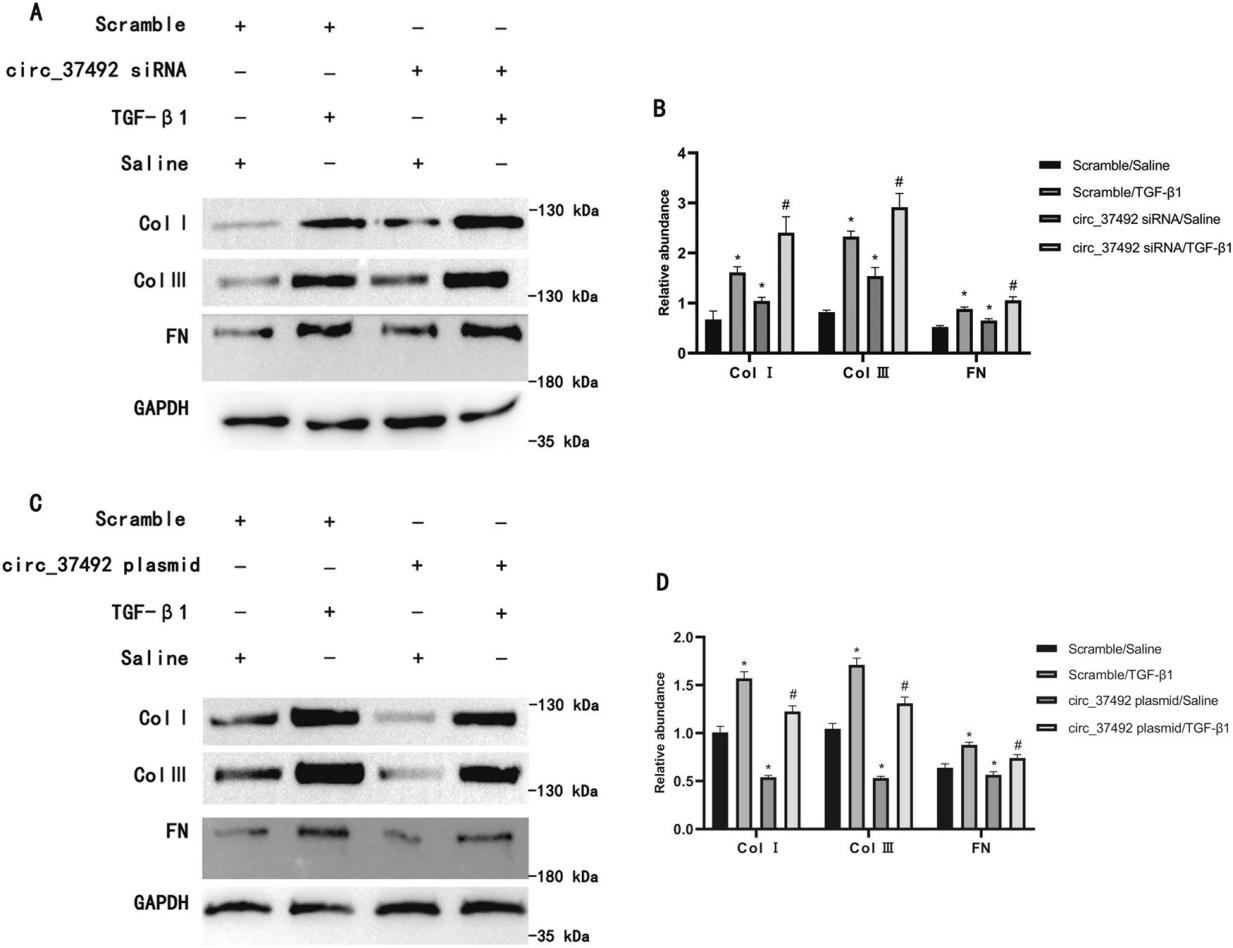
Opposite fibrotic effects in circRNA_37492 inhibition & overexpression. BUMPT cells were pre-transfected with either scrambled siRNA or siRNA circRNA_37492, scramble plasmids, or circRNA_37492 plasmids and co-treated with or without TGF-β1 for 24 h. **A** Type I, III collagen and FN expression by western blot analysis. **B** Quantification of protein band of type I collagen, type III collagen, and FN by grayscale analysis. **C** Type I, III collagen and FN expression by western blot analysis. **D** Quantification of protein band of type I collagen, type III collagen, and FN by grayscale analysis. Quantitative data are presented as the mean ± SD (*n* = 6 per group). *p < 0.05, TGF-β1 or siRNA circRNA_37492 group vs. double control group, TGF-β1 or circRNA_37492 plasmids group vs. double control group; ^#^*p* < 0.05, siRNA circRNA_37492 with TGF-β1 group vs. TGF-β1 group, circRNA_37492 plasmids with TGF-β1 group vs. TGF-β1 group.

To further clarify the role of circRNA_37492 in renal fibrosis, siRNA circRNA_30032 was transfected into BUMPT cells, and then treated with or without TGF-β1 for 24 h. The RT-qPCR analysis showed that siRNA circRNA_30032 silenced the expression of circRNA_30032 under basic and TGF-β1 treatment condition (Fig. S3A). Further western blot results showed that siRNA circRNA_30032 increased expression of type I, III collagen and fibronectin (FN), not only over basal levels but also over those induced by TGF-β1 (Fig. 2A), which supported by grayscale analysis (Fig. 2B).

The above data verified that circRNA_37492 has an anti-fibrosis role, hence, we proposed that overexpression of it might ameliorate renal fibrosis. Here, The RT-qPCR analysis showed that overexpression of circRNA_30032 enhanced the expression of circRNA_30032 under basic and TGF-β1 treatment condition (Fig. S3B). Western blot results showed that circRNA_30032 plasmid treatment decreased expression of type I, III collagen and FN, no matter in basal levels or those induced by TGF-β1 (Fig. 2C), this was confirmed by grayscale analysis (Fig. 2D). Together, these results further confirmed that circRNA_37492 inhibits renal fibrosis.

### CircRNA_37492 directly binds to miR-7682-3p

**Fig. 3:**
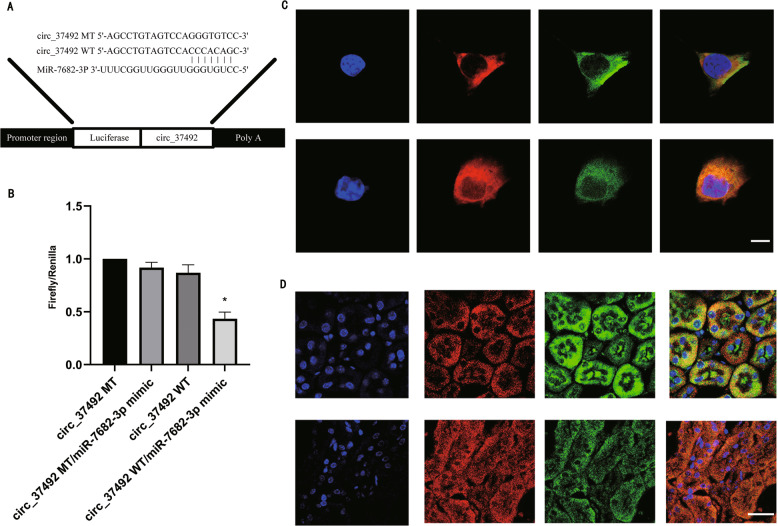
MiR-7682-3p was direct target of circRNA_37492. **A** CircRNA_37492 contained the complementary binding region sequence of miR-7682-3p. **B** After co-transfection with vector of circRNA_37492-WT or circRNA_37492-MT and miR-7682-3p mimic or scramble, the luciferase activities were detected. **C** The co-localization of circRNA_37492 and miR-7682-3p in BUMPT cells treated with TGF-β1. Scale bar, 10 μm. **D** The co-localization of circRNA_37492 and miR-7682-3p in kidney tissue of C57BL/6 mice subjected to UUO. Scale bar, 50μm. Quantitative data are presented as the mean ± SD (*n* = 6 per group). *p < 0.05, circRNA_37492 WT with miR-7682-3p vs. other groups. MT, mutant type; WT, wild type.

The prediction from Arraystar’s home-made miRNA target prediction software showed that circRNA_37492 contained the binding sites of five miRNAs, among them, miR-7682-3p, as a component of the ceRNA network, was a potential downstream target of circRNA_37492 (Fig. 3A). The luciferase reporter assay verified that miR-7682-3p significantly inhibited the luciferase activities of wild-type circRNA_37492, but the luciferase activity of mutant circRNA_37492 was not affected (Fig. 3B). The FISH results indicated that co-location of circRNA_37492 and miR-7682-3p occurred in both the cytoplasm of BUMPT cells and renal tissue of C57BL/6 mice. More than that, the fluorescence intensity of circRNA_37492 was enhanced in parallel with a weakened fluorescence intensity of miR-7682-3p, when given TGF-β1 or UUO treatment (Fig. 3C, D). In addition, RT-qPCR showed that knockdown of circRNA_37492 reversed TGF-β1 induced suppression of miR-7682-3p, while circRNA_37492 overexpression enhanced this effect (Fig. S4A, S4B). Collectively, circRNA_37492 could serve as a miR-7682-3p sponge.

### Upregulation of type I, III collagen and FN with MiR-7682-3p mimic

**Fig. 4:**
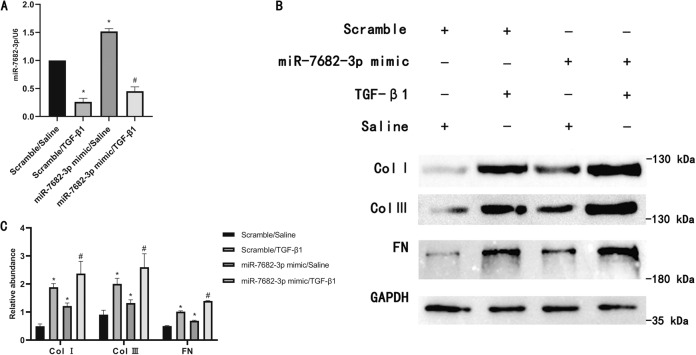
Upregulation of type I, III collagen and FN with MiR-7682-3p mimic. BUMPT cells were pre-transfected with either scramble mimic or miR-7682-3p mimic and co-treated with or without TGF-β1 for 24 h. **A** MiR-7682-3p expression by RT-qPCR analysis. **B** Type I, III collagen and FN expression by western blot analysis. **C** Quantification of protein band of type I collagen, type III collagen, and FN by grayscale analysis. Quantitative data are presented as the mean ± SD (*n* = 6 per group). *p < 0.05, TGF-β1 or miR-96-5p mimic group vs. control group; ^#^*p* < 0.05, miR-7682-3p mimic with TGF-β1 group vs. TGF-β1 group.

MiR-7682-3p was predicted to target 5913 genes with 8734 sites in the 3’UTR, however, its role in renal fibrosis remains unclear. The RT-qPCR analysis indicated that the expression of miR-7682-3p was notably enhanced under basic and TGF-β1 treatment (Fig. 4A). In addition, western blot results showed that miR-7682-3p mimic treatment increased expression of type I, III collagen and FN, not only over basal levels but also over those induced by TGF-β1(Fig. 4B, C). Functionally, miR-7682-3p may played a promoting fibrosis role in BUMPT cells.

### MiR-7682-3p directly target the antifibrotic Fgb

**Fig. 5:**
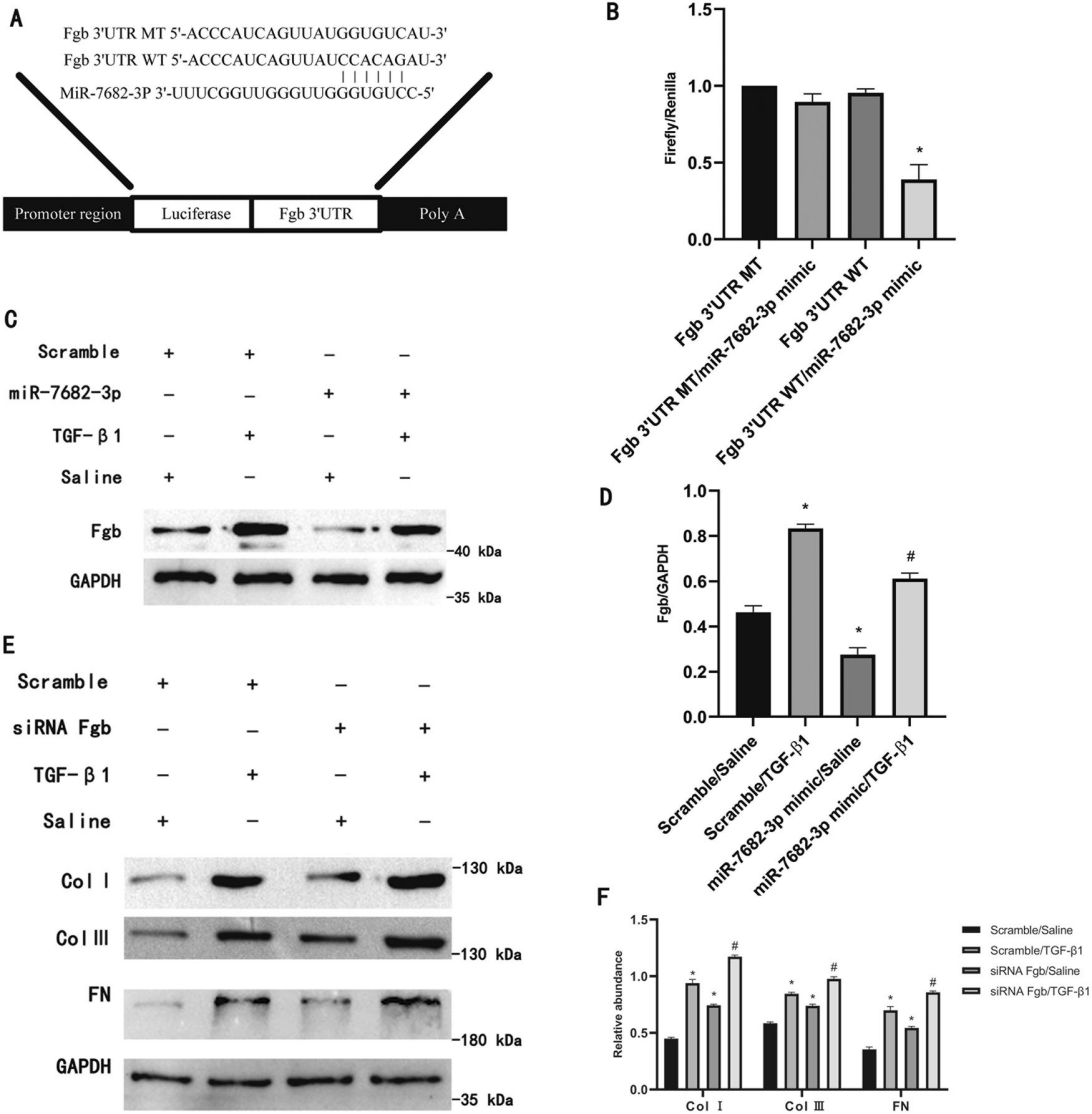
MiR-7682-3p directly target the antifibrotic Fgb. BUMPT cells were pre-transfected with either miR-7682-3p mimic or siRNA Fgb and co-treated with or without TGF-β1 for 24 h. **A** 3’UTR of Fgb mRNA contained the complementary binding region sequence of miR-7682-3p. **B** After co-transfection with vector of Fgb 3’ UTR WT or Fgb 3’UTR MT and miR-7682-3p mimic or scramble, the luciferase activities were detected. **C** Western blot analysis of Fgb. **D** Densitometric analysis of proteins signals. **E** Type I, III collagen and FN expression by western blot analysis. **F** Densitometric analysis of proteins signals. Quantitative data are presented as the mean ± SD (*n* = 6 per group). *p < 0.05, TGF-β1 or miR-7682-3p mimic group vs. control group, TGF-β1 or siRNA Fgb group vs. control group, Fgb 3’UTR WT with miR-7682-3p vs. other groups; ^#^*p* < 0.05, miR-7682-3p mimic with TGF-β1 group vs. TGF-β1 group, siRNA Fgb with TGF-β1 group vs. TGF-β1 group. MT, mutant type; WT, wild type.

Fgb (fibrinogen beta chain gene) can facilitate early wound healing by polymerizing into insoluble fibrin matrix to stabilize the lesion, and promote cell migration and proliferation during re-epithelialization. It was also was involved in anti-liver fibrosis and functioned in protecting against kidney ischemia/reperfusion injury [16–18]. In the preliminary analysis, we identified that Fgb is the potential target gene of miR-7682-3p by TargetScan platform (Fig. 5A). The luciferase reporter assay verified that miR-7682-3p significantly inhibited the luciferase activities of wild-type Fgb 3’UTR, but the luciferase activity of mutant Fgb 3’UTR was not affected (Fig. 5B). In addition, miR-7682-3p mimic treatment attenuated the mRNA and protein expression of Fgb, no matter in basal levels or those induced by TGF-β1 (Figs. S5A, 5C, D). Furthermore, by inhibition of Fgb, we found that siRNA Fgb worked well with or without TGF-β1 treatment (Fig. S5B), and the knockdown of Fgb enhanced the TGF-β1 induced expression of type I, III collagen and FN, however, this effect was reversed by Fgb overexpression (Figs. 5E, F, S5C, S5D), suggesting the antifibrotic effect of Fgb. So we inferred that the antifibrotic Fgb was direct target of miR-7682-3p.

### MiR-7682-3p mediated the antifibrotic effects of circRNA_37492

**Fig. 6:**
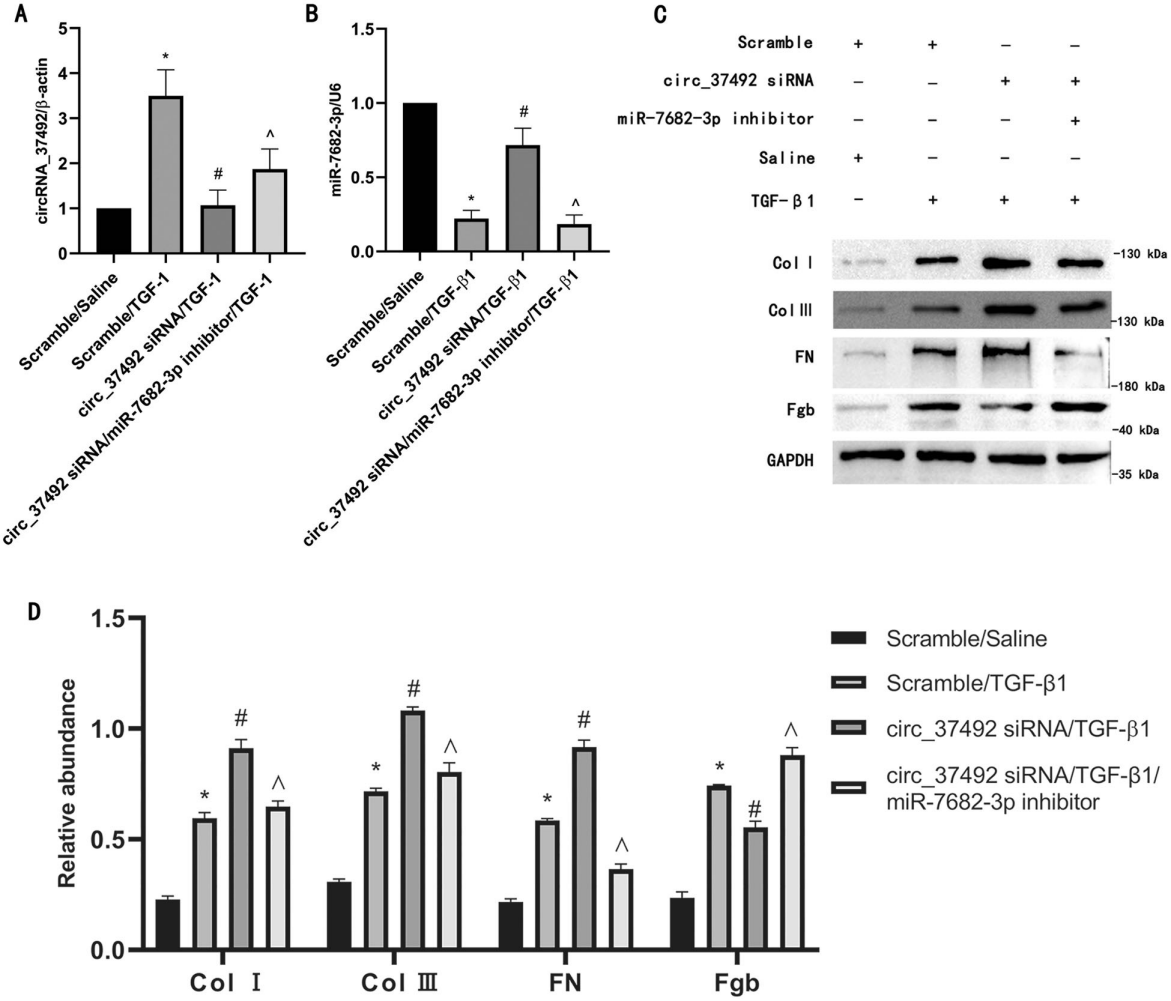
MiR-7682-3p mediated the antifibrotic effects of circRNA_37492. BUMPT cells were pre-transfected with either siRNA circRNA_37492 with miR-7682-3p inhibitor or siRNA circRNA_37492 only and co-treated with TGF-β1 for 24 h. **A** CircRNA_37492 expression by RT-qPCR analysis. **B** MiR-7682-3p expression by RT-qPCR analysis. **C** Type I, III collagen, FN and Fgb expression by western blot analysis. **D** Quantification of protein band of type I collagen, type III collagen, FN, and Fgb by grayscale analysis. Quantitative data are presented as the mean ± SD (*n* = 6 per group). *p < 0.05, TGF-β1 group vs. control group; ^#^*p* < 0.05, siRNA circRNA_37492 with TGF-β1 group vs. TGF-β1 group; ^*p* < 0.05, siRNA circRNA_37492 plus with miR-7682-3p inhibitor and TGF-β1 group vs. siRNA circRNA_37492 with TGF-β1 group.

To further confirm whether antifibrotic role of circRNA_3749 was mediated by the miR-7682-3p, we performed a recovery assay with siRNA circRNA_37492 and miR-7492-3p inhibitor. RT-qPCR showed that they were successfully transfected into BUMPT cells and worked well (Fig. 6A, B). Western blot results indicated that siRNA circRNA_37492 enhanced the expression of type I, III collagen and FN, and attenuated Fgb expression, however, this effect was dramatically reversed by miR-7682-3p inhibitor (Fig. 6C, D). We could therefore confirm that circRNA_37492 protected against renal fibrosis by sponging miR-7682-3p.

### Overexpression of circRNA_37492 attenuated the UUO-induced renal fibrosis via targeting miR-7682-3p/Fgb axis

**Fig. 7:**
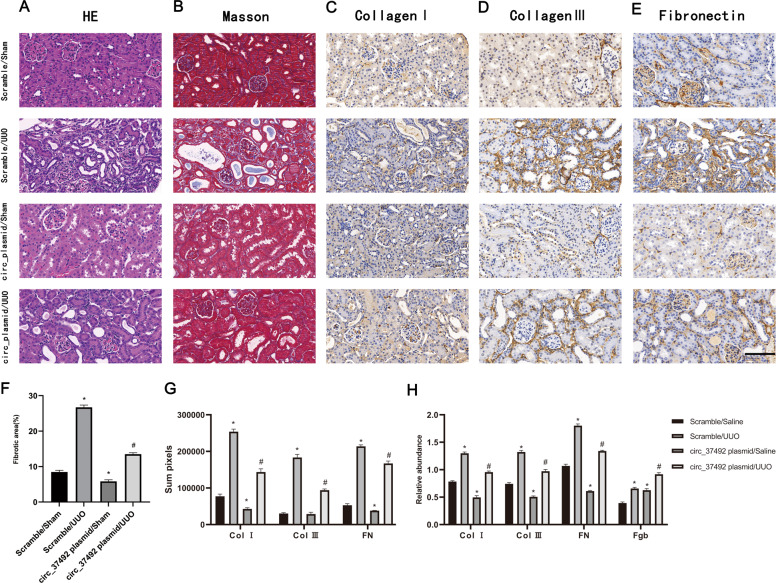
The circRNA_37492 plasmid attenuated the UUO-induced renal fibrosis. C57BL/6 mice (male, 8–10 weeks of age) were injected with 300 μL saline in the absence or presence of 25 µg circ_37492 plasmid and then subjected to UUO or sham surgery. **A**–**E** The HE staining (**A**), Masson staining (**B**), and immunohistochemical staining of collagen I (**C**), collagen III (**D**), fibronectin (**E**) of renal tissue slices. (**F**) Quantification of fibrotic area (%) in the kidney cortex. **G** Quantification of immunohistochemical staining of type I collagen, type III collagen, and FN. **H** Quantification of protein band of type I collagen, type III collagen, FN, and Fgb in western blot. Original magnification, ×400. Scale bar, 100 μm. Quantitative data are presented as the mean ± SD (*n* = 6 per group). *p < 0.05, circRNA_37492 plasmid group or UUO group vs. double control group; ^#^*p* < 0.05, circRNA_37492 plasmid with UUO group vs. UUO group.

To further explore the antifibrotic role of circRNA_37492 in vivo, circRNA_37492 plasmids were injected via tail vein, and then subjected to UUO as described above. In corresponding with the experiment in vitro, overexpression of circRNA_37492 attenuated UUO-induced tubular dilation, renal cortical atrophy, and ECM accumulation on HE and Masson’s trichrome staining (Fig. 7A, B, F), which were further confirmed by immunostaining of type I, III collagen and FN (Fig. 7C–E, G). Furthermore, it is confirmed that UUO treatment would upregulate circRNA_37492 and downregulate miR-7682-3p in mice kidney, and this effect would be further enhanced by tail vein injection of circRNA_37492 plasmid (Fig. S6A, S6B). Besides, the western blot also indicated that mice subjected to UUO would be protected from accumulation of type I, III collagen, and FN in kidney and highly express Fgb in kidney by receiving circRNA_37492 plasmid treatment (Figs. 7H, S6C). Based upon the collective results, we could conclude that circRNA_37492 protected against renal fibrosis via targeting miR-7682-3p/Fgb axis in vitro and vivo.

### Homologous hsa_circRNA_0012138 as potential target in human renal fibrosis

**Fig. 8:**
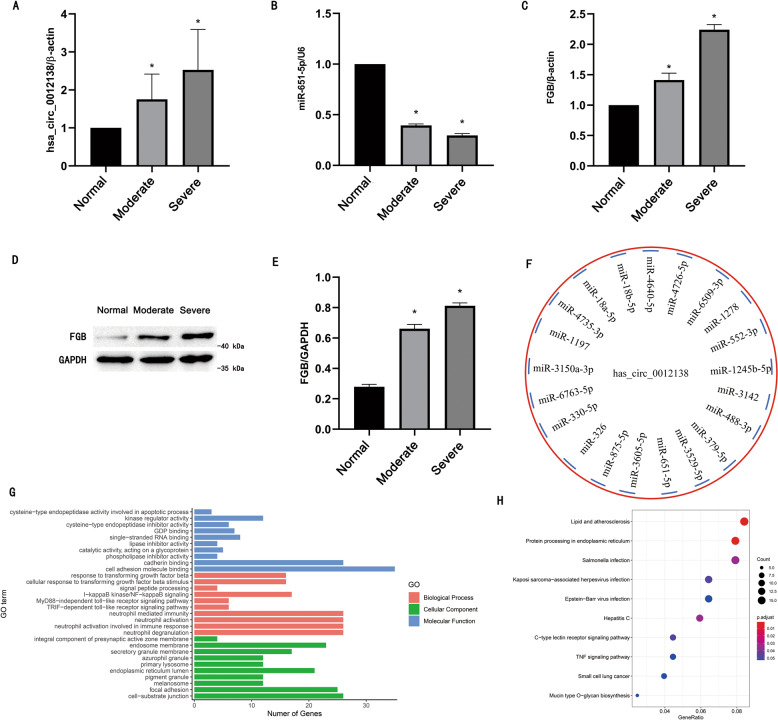
Homologous hsa_circRNA_0012138 as potential target in human renal fibrosis. **A** Hsa_circRNA_0012138 expression by RT-qPCR analysis. **B** Hsa_miR-651-5p expression by RT-qPCR analysis. **C** FGB expression by RT-qPCR analysis. **D** FGB expression by western blot analysis. **E** Quantification of protein band of FGB by grayscale analysis. **F** MicroRNAs predicted to interact with hsa_circRNA_0012138. **G** Gene Ontology enrichment analysis in microRNAs targeted genes. **H** Kyoto Encyclopedia of Genes and Genomes enrichment analysis in microRNAs targeted genes. Samples were obtained from postoperative specimens of three group of patients. Normal: carcinoma-adjacent normal tissues of patients undergoing radical nephrectomy; Moderate group: obstructive hydronephrosis kidney tissues of patients undergoing hydronephroctomy; Severe group: obstructive hydronephrosis kidney tissues of patients undergoing hydronephroctomy, with longer obstruction time, less unilateral residual GFR and thinner cortical thickness than Group A. *p < 0.05, Severe group or Moderate group vs. Normal group.

hsa_circ_0012138 originates from the best transcript (NM_024066) of ERI3 (gene symbol) and was matched with circRNA_37492 (NM_080469, Eri3) to be the homologous circRNA (Table S1). Eri3 gene can be expressed in kidney and involved with diverse metabolic processes [19]. We further examined the expression of hsa_circ_0012138 in renal cortex from patients with obstructive hydronephrosis, and the general patient characteristics is shown in Table S2. RT-qPCR showed that hsa_circ_0012138 was highly expressed in obstructive hydronephrosis kidney and the expression level increased with the degree of obstruction (Fig. 8A). Besides, 21 miRNAs were predicted to interact with hsa_circ_0012138 (Fig. 8F) and 640 upregulated genes were predicted to be target of these miRNAs (Table S3). Among them, miR-651-5p and its target FGB (human fibrinogen beta chain gene) were of low expression and high expression, respectively (Fig. 8B–E), this change was further increased with the worsening of obstruction. The data above suggested that hsa_circ_0012138/miR-651-5p/FGB may constitute potential ceRNA axis in human ON. Moreover, gene ontology functional enrichment among the 640 genes revealed that they were mainly enriched in terms of “transforming growth factor beta”, “apoptotic process”, “NF-kappa B signaling” and “toll-like receptor signaling pathway”, “kinase regulator activity” and “cell adhesion” (Fig. 8G), which were previously demonstrated to related to renal fibrosis or ECM deposition [20–22]. KYOTO ENCYCLOPEDIA OF GENES AND GENOMES pathway enrichment among these genes showed that they were mainly enriched in terms of “TNF signaling pathway”, “Hepatitis” and “virus infection” (Fig. 8H), which were also reported to related to renal fibrosis or ECM deposition [23, 24]. Collectively, these results indicated that hsa_circ_0012138 could be involved in human renal fibrosis by sponging various miRNAs.

## Discussion

As a result of the special loop structure, circRNAs are more stable than their linear host genes, so multiple sequence-conserved mouse circRNAs may exist in human. The role and regulatory mechanism of circRNAs in renal fibrosis need urgently to be uncovered. In this study, by construction of the ceRNA network, we for the first time identified a mouse circRNA with its homologous hsa_circ_0012138 associated with the pathophysiology of renal fibrosis. Mechanistically, circRNA_37492 bound to miR-7682-3p as its ceRNA to induce the expression of Fgb. Interestingly, overexpression of circRNA_37492 attenuated the TGF-β1/UUO-induced renal fibrosis via targeting the miR-7682-3p/Fgb axis.

Several studies reported that circRNAs mediated the progression of diabetic nephropathy, lupus nephritis, and focal segmental glomerulosclerosis [8–15], and only one study demonstrated that circRNA_30032 promoted fibrosis in UUO mice kidney [25]. In the present study, topological analyses based on the ceRNA network suggested that circRNA_37492 with a homologous hsa_circ_0012138 was the most potential target, considering the fold change and conservation score. To the knowledge, exonic circRNAs are mainly located in the cytoplasm [26], consistently, we found that circRNA_37492 was significantly induced in the cytoplasm of BUMPT cells by TGF-β1. In addition, endogenous circRNA_37492 exerts an antifibrotic potential for the results shown in Figs. 2 and 7.

As we know, circRNAs usually sponged miRNA to regulate mRNA [27–31]. Here, we validated that circRNA_37492 sequence harbored binding site for miR-7682-3p, which suggested miR-7682-3p as a putative target of circRNA_37492, and deduced that that miR-7682-3p is a direct target of circRNA_37492 (Figs. 3, S4). Our data for the first time reported that miR-7682-3p promoted TGF-β1 induced renal fibrosis. To further investigate the mechanism of miR-7682-3p, we found that Fgb was a target of it (Figs. 5, S5). In the present study, TGF-β1/UUO-induced the increasing of Fgb in mice proximal tubular cells, and we demonstrated that Fgb suppressed the renal fibrosis (Figs. 5, S5, S6). However, there were few literatures on the anti-kidney fibrosis function of Fgb. Previous literature reported that Fgb protein is primarily located in the renal interstitium, and Fgb-derived peptide protects mice from I/R-induced kidney injury by aiding in epithelial cell proliferation and tissue repair [18]. Hence, we assumed that interstitial Fgb would not only inhibit tubular cells atrophy, but their transformation into myofibroblast, so as to suppress fibrosis production of myofibroblast. Moreover, the recovery assay shown in Fig. 6 demonstrated that circRNA_37492/miR-7682-3p/Fgb axis has a potent anti-fibrosis nature. Of note, our data only conclude that the regulation pathway might happened in renal tubular cells (Figs. S2, S7), however, it could not rule out the possibility that this happened in fibroblast, vascular endothelial cell, et al.

Interestingly, hsa_circ_0012138/miR-651-5p/FGB may constitute potential ceRNA axis in human renal fibrosis, according to further study (Fig. 8). Not only that, GO and KYOTO ENCYCLOPEDIA OF GENES AND GENOMES enrichment revealed that hsa_circ_0012138 target genes were previously demonstrated to related to renal fibrosis or ECM deposition [20–24]. Thus, these results indicated that hsa_circ_0012138 could be involved in human renal fibrosis by sponging various miRNAs. However, some limitations remain in the present study. First, in consideration of the time and resource consuming, the interactions among hsa_circ_0012138, miRNAs and mRNAs were not further validated by the binding assay, so the hsa_circ_0012138/miR-651-5p/FGB axis need to be further validated by additional experiments. Second, we suspected so far that hsa_circ_0012138 regulated expression of multiple genes by targeting different miRNAs through bioinformatics methods, however, the most significant axis (es) remains unclear and need further validation.

Collectively, our present study found that circRNA_37492 played an anti-fibrosis role in vitro and vivo via targeting miR-7682-3p/Fgb axis. Moreover, we for the first time found that homologous hsa_circRNA_0012138 may function as a ceRNA to regulate multiple gene expressions and is involved in human renal fibrosis. Thus, the data suggest that circRNA_37492/hsa_circ_0012138 may serve as novel therapy target for obstructive renal fibrosis disease.

## Materials and methods

### Construction of the ceRNA regulatory network

The circRNA chip assay was used to detect expression of circRNA in kidney of UUO model. Differential expression analysis was performed for the identification of upregulated circRNAs (fold change >2). Predicted miRNAs were determined using Arraystar’s home-made miRNA target prediction software based on TargetScan (http://www.targetscan.org/) & miRanda (http://www.miranda.org/) [32]. Expression profiles of microRNA were obtained from Gene Expression Omnibus dataset (GSE42716, GSE162794) and differential expression analysis was conducted to select downregulated miRNAs in UUO using ‘limma’ R package (adjusted *P* value < 0.05, |log2FC|> = 1). The predicted microRNAs were intersected with downregulated microRNAs, and then intersection was taken as candidate circRNAs-miRNAs. Next, the targeted genes of candidate miRNAs were predicted using TargetScan database and were intersected with upregulated genes in GSE145053 (adjusted *P* value < 0.01, |log2FC|> = 4) as candidate miRNAs-mRNAs. Finally, the candidate circRNAs-miRNAs were intersected with candidate miRNAs-mRNAs to establish the circRNA-miRNA-mRNA ceRNA network, which was visualized by “ggalluvial” R package. In addition, the NetworkAnalyzer plug-in in Cytoscape software (http://www.cytoscape.org) was used to calculate the topological parameters of the network [33], the circRNAs-miRNAs-mRNAs were screened according to connection degree and fold change. To evaluate the stability of ceRNA network, correlation analysis between the expression levels of circRNAs and mRNAs targeted by miRNAs in the ceRNA network was performed [34].

### Cell culture and treatments

The BUMPT cells were initially obtained from Drs. John Shwartz & William Lieberthal at Boston University [35], and incubated in Dulbeccos modified Eagles medium (Thermo Fisher Scientific) containing 10% fetal bovine serum and 1% penicillin–streptomycin at 37 °C in 5% CO_2_/95% air, and subsequently transfected with miR-7682-3p inhibitor (100 nM), miR-7682-3p mimics (100 nM), circRNA_37492 siRNA (50 nM), circRNA_37492 plasmids (50 nM), siRNA Fgb (100 nM), or negative-control plasmid (Ruibo Biotechnology, Guangzhou, China) using Lipofectamine 2000 Transfection Reagent (Invitrogen, USA). Twenty-four hours after transfection, the cells were starved and treated with or without 5 ng/mL of TGF-β1 (Proteintech, Rosemont, IL, USA) for different times.

### Luciferase reporter assays

The dual luciferase assay kit (cat. no. KGAF040) was purchased from KeyGEN BioTECH (Nanjing, Jiangsu, China), and all the plasmids were constructed by Sangon Biotech Company (Shanghai, China). Reporter assays were performed using the dual luciferase assay system as described previously [36, 37]. The luciferase reporter constructs were generated from the miRNA target expression vector pmirGLO (Promega, Madison, WI, USA). Full-length binding-site-mutant and wild-type circRNA_37492 constructs, truncated mutant and wild-type 3’UTR of fibrinogen beta chain (Fgb) gene were individually co-transfected with miR-7682-3p mimics or Control into BUMPT cells for 48 h. Luciferase activities were then measured using a Synergy™ HTX multi-mode reader (Biotek, Winooski, VT, USA) and normalized according to renilla luciferase activity.

### Animal models

Male C57BL/6 mice (8–10 weeks of age) were purchased from Sippr-BK Laboratory Animal Corporation (Shanghai, China). Animal experiments were reviewed and approved by the Animal Ethical and Welfare Committee of The Second Xiangya Hospital (China). Mice were bred with free water and food in a specific pathogen-free conditions under a 12-h light/12-h dark cycle. The sample size was estimated to 12 (*n* = 3 per group). The mice were randomly allocated to experimental groups by Random function in Excel software (version 2019, Microsoft Corporation) and no blinding was needed. The mice were injected with saline or 25 µg circ_37492 plasmid (Genepharma, Shanghai, China) by tail vein (once a day for consecutive 3 days), and then the left ureter was ligated to construct the UUO model according to the previous studies [38, 39].

### Histology, immunohistochemistry, and immunoblot analyses

Antibodies for Collagen I (cat. no. ab34710), III (cat. no. ab7778), FN (cat. no. ab2413), and Fgb (cat. no. ab189490) were purchased from Abcam (Cambridge, MA, USA), whereas anti-GAPDH (cat. no. T0004) were obtained from Affinity Biosciences (Xiangtai Biological Technology, Changzhou, Jiangsu, China). Mice kidney tissues were fixed and cut into slices, then stained with hematoxylin and eosin (H&E) and Masson’s trichrome as we previously described [40]. We used Image-Pro Plus software (version 6.0, Media Cybernetics, USA) to calculate the area of interstitial fibrosis and gross interstitial, and defined the fibrotic area (%) as percent of interstitial fibrosis versus gross interstitial [40]. The antibodies used in immunohistochemical analysis were as follows: anti-collagen I (dilution 1:100), collagen III (dilution 1:100), FN (dilution 1:200) according to the previous protocol [41]. Immunohistochemistry images were also processed with Image-Pro Plus software (version 6.0, Media Cybernetics, USA), deposition of type I, III collagen, and FN were quantified by calculating optical density [42]. Stained sections were scanned with Pannoramic Desk powered by Pannoramic Digital Slide Scanner (3D Histech, Budapest, Hungary). Snapshots of the slide-scans were taken using CaseViewer software (3D Histech, Budapest, Hungary). For immunoblot analyses, protein samples (20 μg/lane) from BUMPT cells or kidneys were subjected to 8% denaturing PAGE (SDS-PAGE) [43–45], and then transferred onto a Polyvinylidene difluoride membrane (Millipore, Bedford, MA, USA). The membrane was incubated with specific primary antibodies against collagen I, collagen III, FN, Fgb, and GAPDH, followed by incubation with the secondary antibody (Servicebio, China) according to standard protocol. Protein signal was examined with the Tanon-5200Multi electroluminescence detection system (Tanon Science & Technology, Shanghai, China) and quantified using ImageJ software (version 1.8.0, https://imagej.nih.gov/ij/) [46].

### Real-time qPCR

All PCR primers were synthesized by Sangon Biotech Company (Shanghai, China) and listed in Table S4. Total RNA was extracted from BUMPT cells, kidney tissue of C57BL/6J mice and kidney tissue of patients by using Trizol reagent (Invitrogen, Carlsbad, CA, USA), and then reverse-transcribed to cDNA. The expression of circRNA, miRNA and mRNA were detected by the RT-qPCR assay on a LightCycle96 RT-qPCR system (Roche Applied Science, Mannheim, Germany) using SYBR Green Premix Pro Taq HS qPCR Kit (cat. no. AG11701, AG Bio, Changsha, Hunan, China) according to the manufacturer’s instructions. The sequences of miR-7682-3p were obtained in the miRDB database. The sequences of hsa_circ_0012138 were obtained in the circBase database. The sequences of circRNA_37492, which was derived from the NM_080469 (gen ID: 140546), were provided in Table S5. β-Actin was used as the internal reference of CircRNA and mRNA, and miRNA employed U6 as internal reference. Relative quantification was carried out using the comparative ΔCq method.

### Fluorescence in site hybridization

FISH Kit (cat. no. C10910) was purchased from RiboBio, RNA FISH probes were also synthesized by RiboBio (Guangzhou, China). The experiment was performed based on the instructions of the FISH Kit. For FISH analysis, BUMPT cells and mice kidney were fixed with 4% paraformaldehyde (Sigma) and hybridized with the fluorescence probes of miR-7682-3p and circRNA_37492. U6 and 18S rRNA served as the nuclear and cytoplasmic controls, respectively. DAPI was used to stain nuclei, U6, 18S rRNA, and circRNA37492 were labeled by CY3, miR-7682-3p was labeled by FAM. The slides were hybridized at 37 °C overnight with the probes. The confocal Laser Scanning Microscope (LSM780NLO, Carl Zeiss, Germany) was used to take fluorescence images.

### Human samples

The Ethics Committee of The Second Xiangya Hospital, Central South University approved the research use of human samples. Written informed consent was also obtained from all participants. Patients with the age less than 18, presence of other kidney diseases were excluded. Human kidney samples were collected from patients undergoing hydronephroctomy (*n* = 8) and radical nephrectomy (*n* = 4) at the clinic (Fig. S8). Western blot and RT-qPCR method were employed to quantify the expression of key ceRNA molecules in human kidneys.

### Gene function and relevant pathway enrichment analysis

Sequence conservation for circRNA_37492 was tested by blast function in circBase website (http://circrna.org/cgi-bin/webBlat), as a result, hsa_circ_0012138 was matched with it to be the homologous circRNA, and was further tested expression levels in human obstructive hydronephrosis kidney samples by RT-qPCR. miRNAs interacting with hsa_circ_0012138 were predicted based on the starBsae (http://starbase.sysu.edu.cn/) [47, 48]. On the one hand, mRNAs targeted by predicted miRNAs were retrieved based on the TargetScan (http://www.targetscan.org/), on the other hand, differential expression analysis were performed to identify upregulated genes based on gene expression profiles in GSE66494 dataset (CKD vs. Control, Homo sapiens) using the R package ‘limma’ (adjusted *P* value < 0.05, |log2FC|> = 1). Only those predicted genes truly upregulated in GSE66494 can be recognized as targeted genes. Accordingly, the expression of homologous gene and its predicted binding miRNA were assayed by western blot and RT-qPCR. Moreover, to further clarify the potential biological process and understand the potential pathways of differential expression mRNAs regulated by hsa_circ_0012138, we performed Gene ontology and Kyoto Encyclopedia of Genes and Genomes enrichment analysis using the R package ‘clusterProfiler’, and then visualized the top 10 terms [49, 50].

### Statistical analyses

Statistical analyses were performed using GraphPad Prism (version 9.12, San Diego, CA, USA). For comparisons between two groups, we used two-tailed Student *t*-tests, for multiple group comparison, we used one-way ANOVA test. Quantitative data are presented as the mean and SD (mean ± SD). The difference was considered statistically significant when *P* < 0.05.

## Supplementary information

Supplementary Information

Original Data

aj-checklist

## Data Availability

The datasets used and/or analyzed during the current study are available from the corresponding author on reasonable request.
