# Supplementary material for: The mmu_circRNA_37492/hsa_circ_0012138 function as potential ceRNA to attenuate obstructive renal fibrosis
Source: Cell Death Dis. 2022 Mar 4;13(3):207. doi: 10.1038/s41419-022-04612-3 (PMC8897503; doi:10.1038/s41419-022-04612-3)
Supplement: Supplementary file 1 — Supplementary Information [file 41419_2022_4612_MOESM1_ESM.pdf]

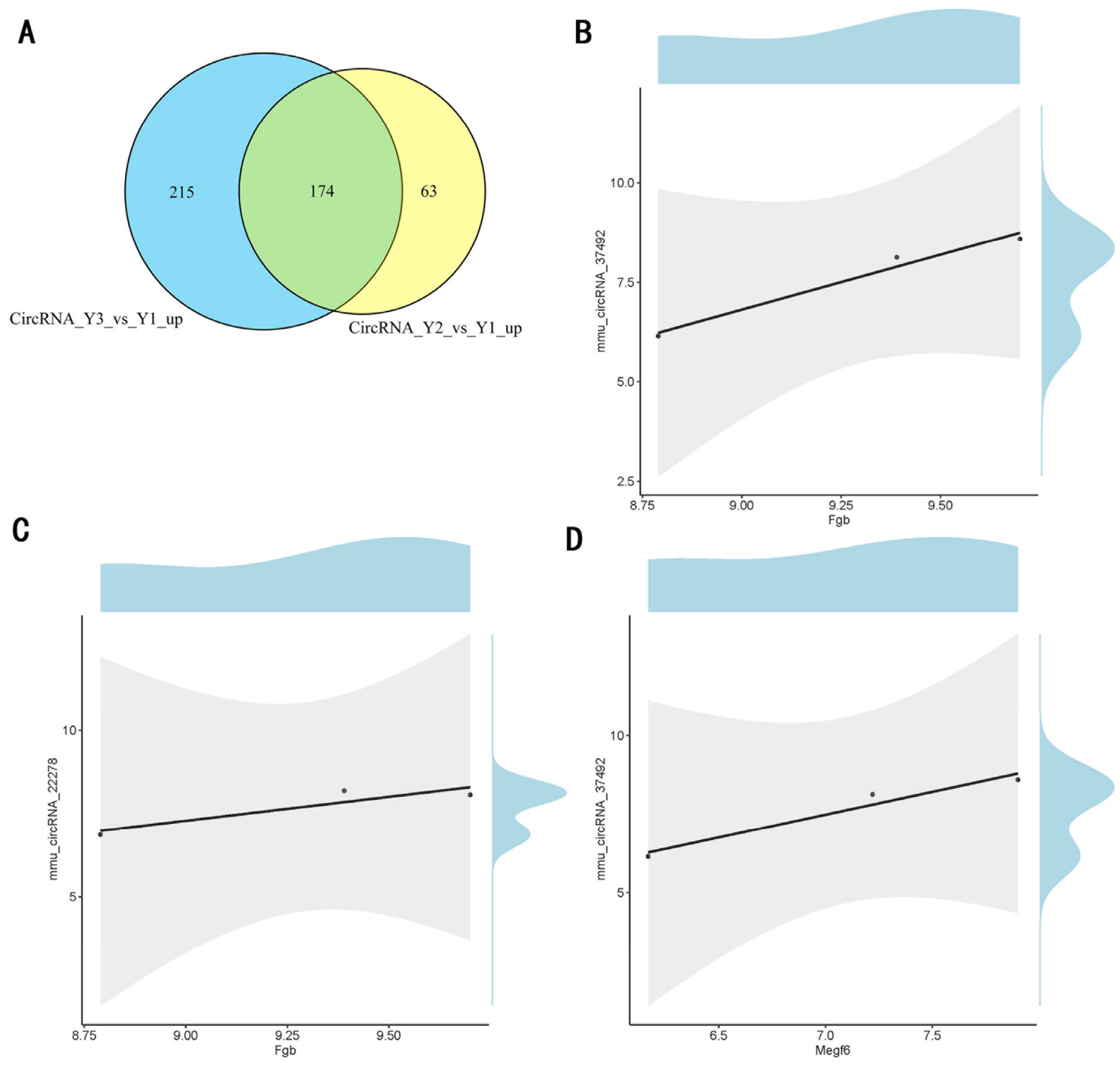

Figure S1. Construction of a circular RNA-associated ceRNA regulatory network in C57BL/6 mice UUO model. (A) The co-upregulation amount of circRNAs at days 3 and 7 in UUO group vs. Sham group (more than 2 fold changes). **(B-D)** Regression analysis between the expression levels of differentially expressed circRNAs and differentially expressed mRNAs targeted by miR-7682-3p in the ceRNA network. The horizontal axis represents the differentially expressed mRNAs level, and the vertical axis indicates the differentially expressed circRNAs level. The upper and right edges are waveform graphs of gene expressions.

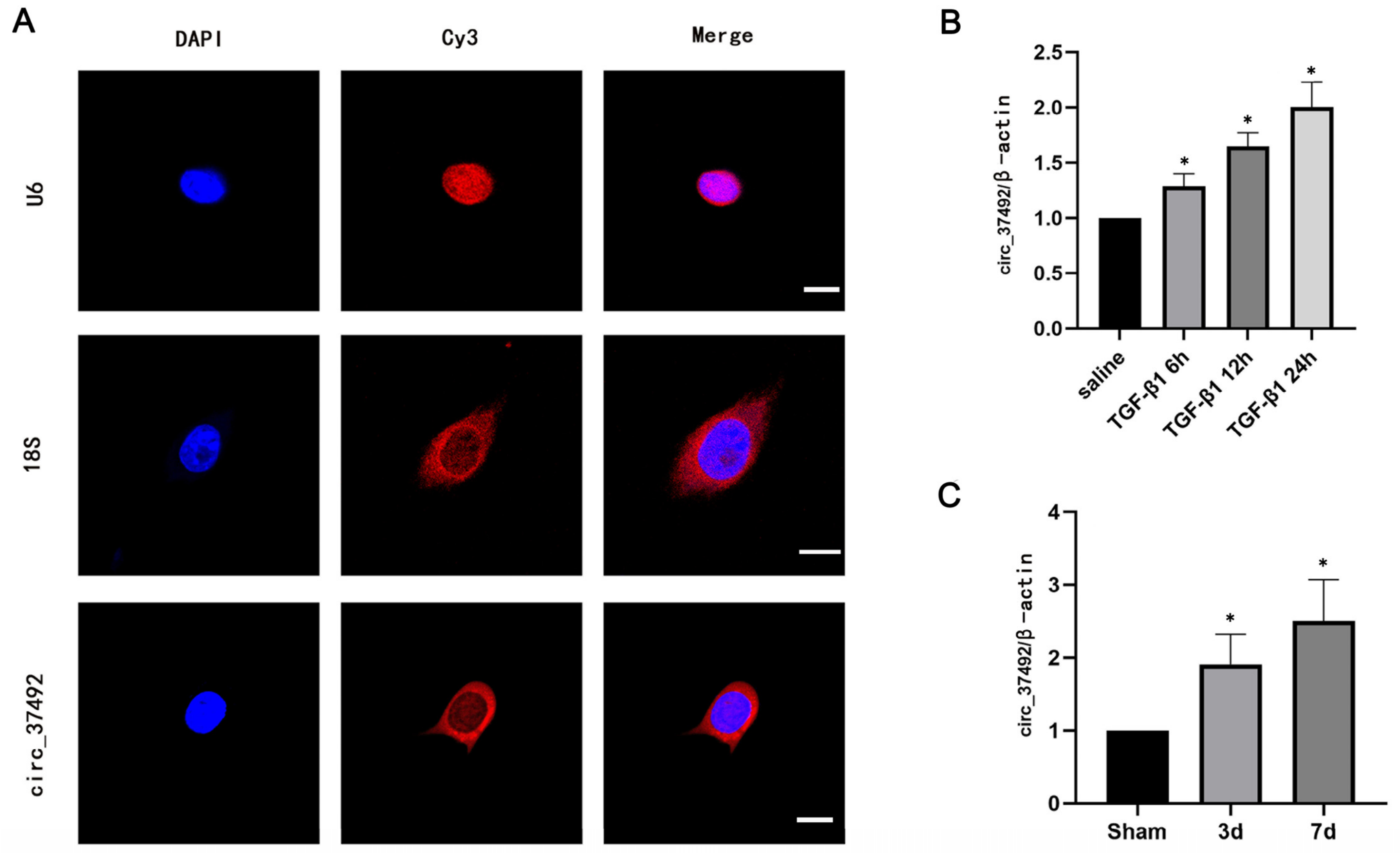

Figure S2. Induction of circRNA_37492 in TGF-β1 treated BUMPT cells and UUO mice. BUMPT cells were treated with 5ng/ml TGF-β1 at indicated time points. C57BL/6 mice were subjected to the UUO for 3 and 7 days. **(A)** RNA FISH analysis of circRNA_37492 localization in BUMPT cells. Scale bar, 10μm **(B)** RT-qPCR analysis of the expression levels of circRNA_37492 in TGF-β1 treatment at 6h, 12h, 24h. **(C)** RT-qPCR analysis of the expression levels of circRNA_37492 in UUO at days 3 and days 7. Data are expressed as mean ± SD (n = 6). * One-way ANOVA test $p<0.05$, TGF-β1 treatment at 6h, 12h, 24h versus saline control, UUO for 3 and 7 days versus sham surgery control.

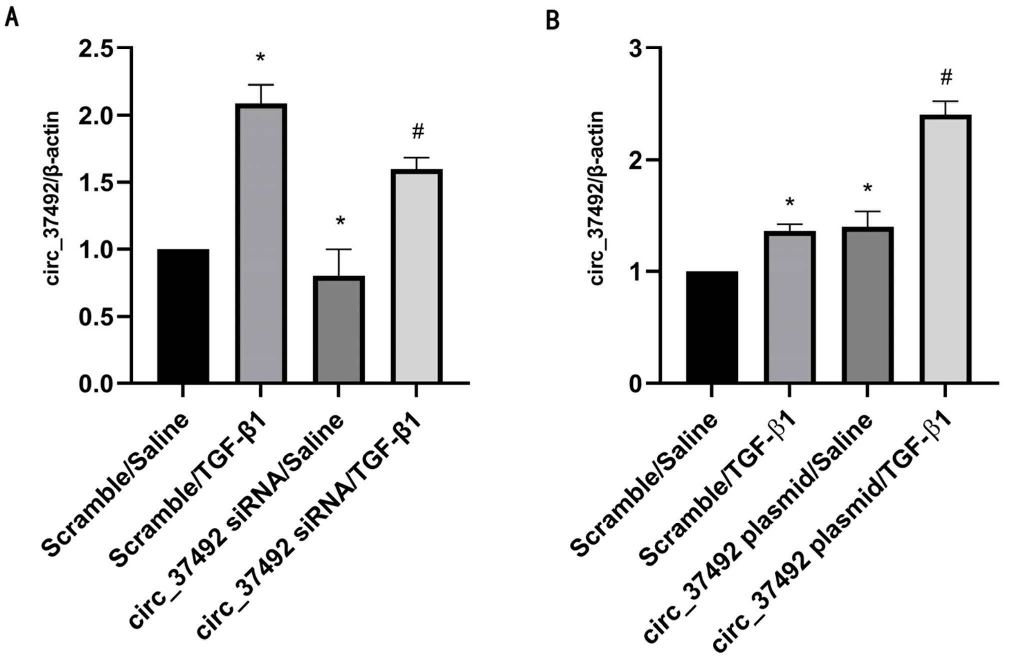

Figure S3. circRNA_37492 inhibition and overexpression. BUMPT cells were pre-transfected with either scramble siRNA or siRNA circRNA_37492 and co-treated with or without TGF-β1 for 24 h. **(A)** CircRNA_37492 expression by RT-qPCR analysis. Quantitative data are presented as the mean ± SD (n = 6 per group). * p<0.05, TGF-β1 or siRNA circRNA_37492 group vs. double control group; # p<0.05, siRNA circRNA_37492 with TGF-β1 group vs. TGF-β1 group.

BUMPT cells were pre-transfected with either scramble plasmids or circRNA_37492 plasmids and co-treated with or without TGF-β1 for 24 h. **(B)** CircRNA_37492 expression by RT-qPCR analysis. Quantitative data are presented as the mean ± SD (n = 6 per group). * p<0.05, TGF-β1 or circRNA_37492 plasmids group vs. double control group; # p<0.05, circRNA_37492 plasmids with TGF-β1 group vs. TGF-β1 group.

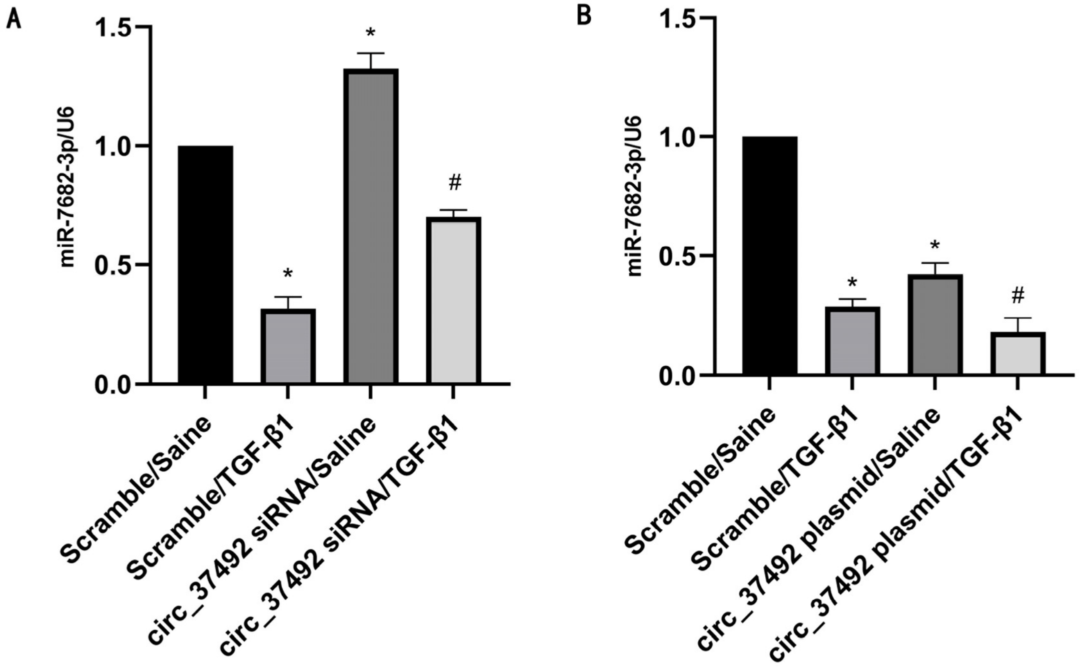

Figure S4. MiR-7682-3p was direct target of circRNA_37492. (A) Detection of miR-7682-3p expression in BUMPT cells transfected with siRNA circRNA_37492 by RT-qPCR analysis. **(B)** Detection of miR-7682-3p expression in BUMPT cells transfected with circRNA_37492 plasmids by RT-qPCR analysis. * $p<0.05$, TGF-β1 or siRNA circRNA_37492 group vs. control group, TGF-β1 or circRNA_37492 plasmid group vs. control group; # $p<0.05$, siRNA circRNA_37492 or circRNA_37492 plasmids with TGF-β1 group vs. TGF-β1 group.

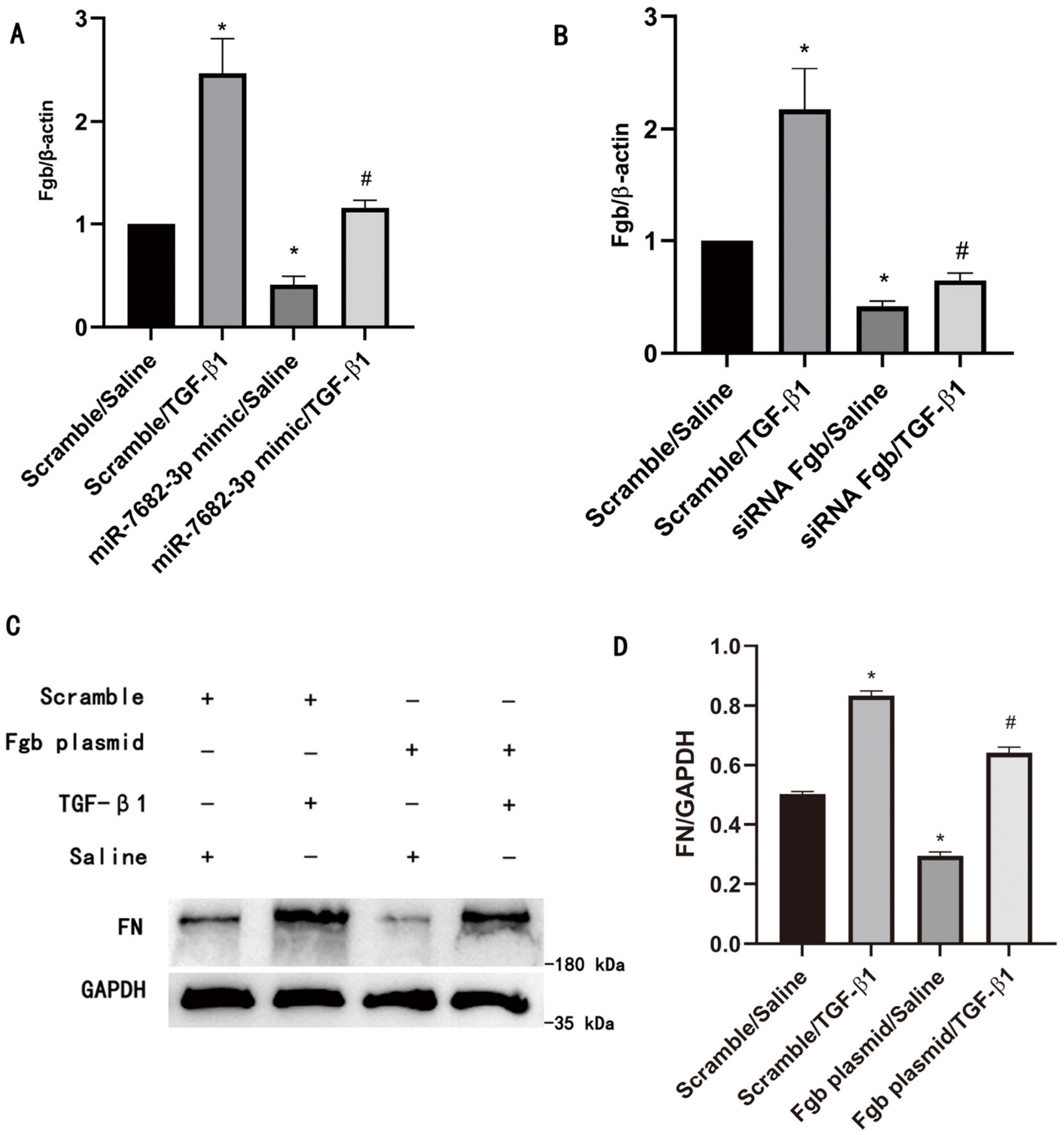

Figure S5. MiR-7682-3p directly target the antifibrotic Fgb. BUMPT cells were pre-transfected with either miR-7682-3p mimic or siRNA Fgb or Fgb plasmid and co-treated with or without TGF-β1 for 24 h. **(A)** RT-qPCR analysis of Fgb. **(B)** RT-qPCR analysis of Fgb. **(C)** FN expression by western blot analysis. **(D)** Densitometric analysis of proteins signals. Quantitative data are presented as the mean ± SD (n = 6 per group). * $p < 0.05$, TGF-β1 or miR-7682-3p mimic group vs. control group, TGF-β1 or siRNA Fgb group vs. control group, TGF-β1 or Fgb plasmid group vs. control group; # $p < 0.05$, miR-7682-3p mimic with TGF-β1 group vs. TGF-β1 group, siRNA Fgb with TGF-β1 group vs. TGF-β1 group, Fgb plasmid with TGF-β1 group vs. TGF-β1 group.

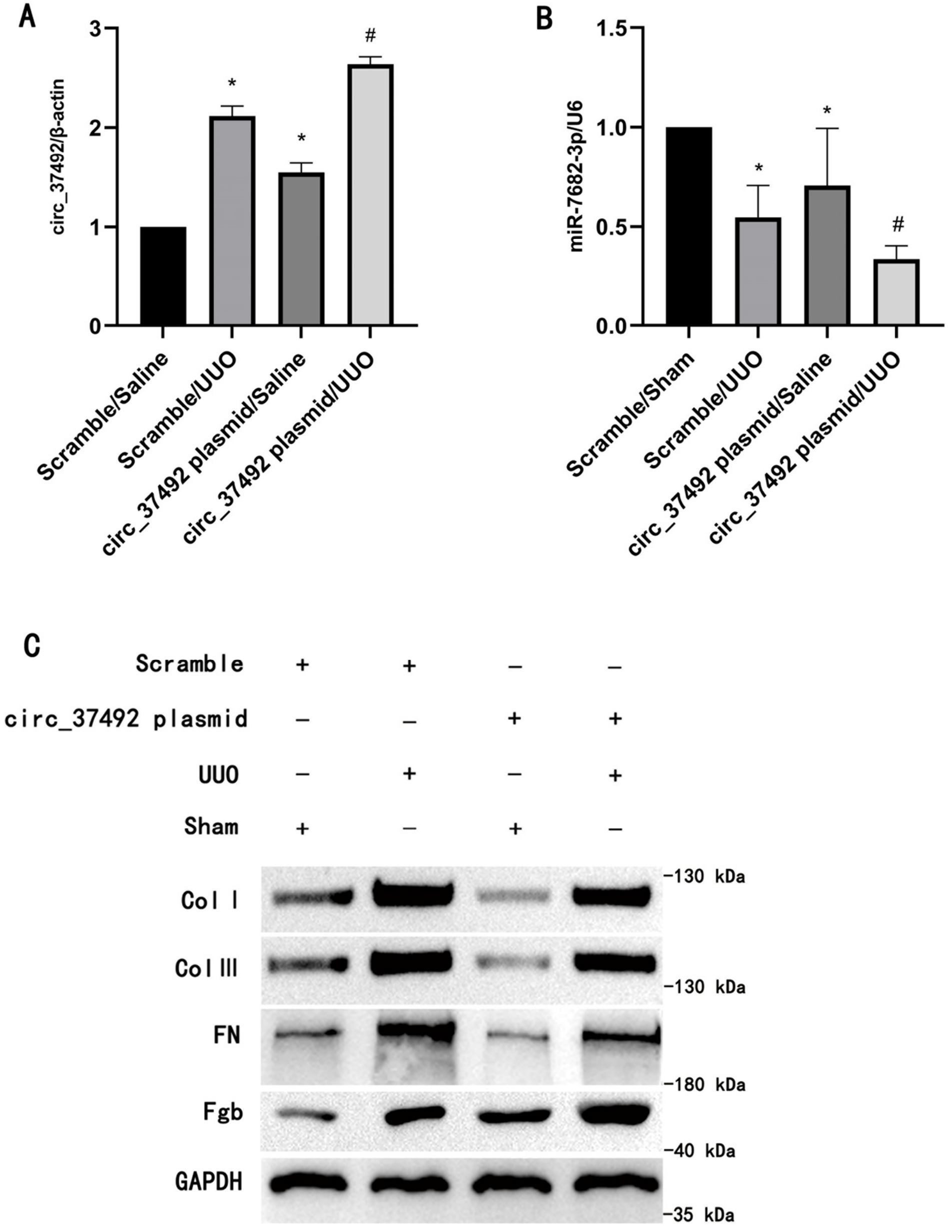

Figure S6. Overexpression of circRNA_37492 attenuated the UUO induced renal fibrosis via targeting miR-7682-3p / Fgb axis. C57BL/6 mice (male, 8-10 weeks of age) were injected with 300μL saline in the absence or presence of 25 μg circ_37492 plasmid and then subjected to UUO or sham surgery. **(A)** CircRNA_37492 expression by RT-qPCR analysis. **(B)** MiR-7682-3p expression by RT-qPCR analysis. **(C)** Type I, III collagen, FN and Fgb expression by western blot analysis. Quantitative data are presented as the mean ± SD (n = 6 per group). * $p < 0.05$, circRNA_37492 plasmid group or UUO group vs. double control group; # $p<0.05$, circRNA_37492 plasmid with UUO group vs. UUO group.

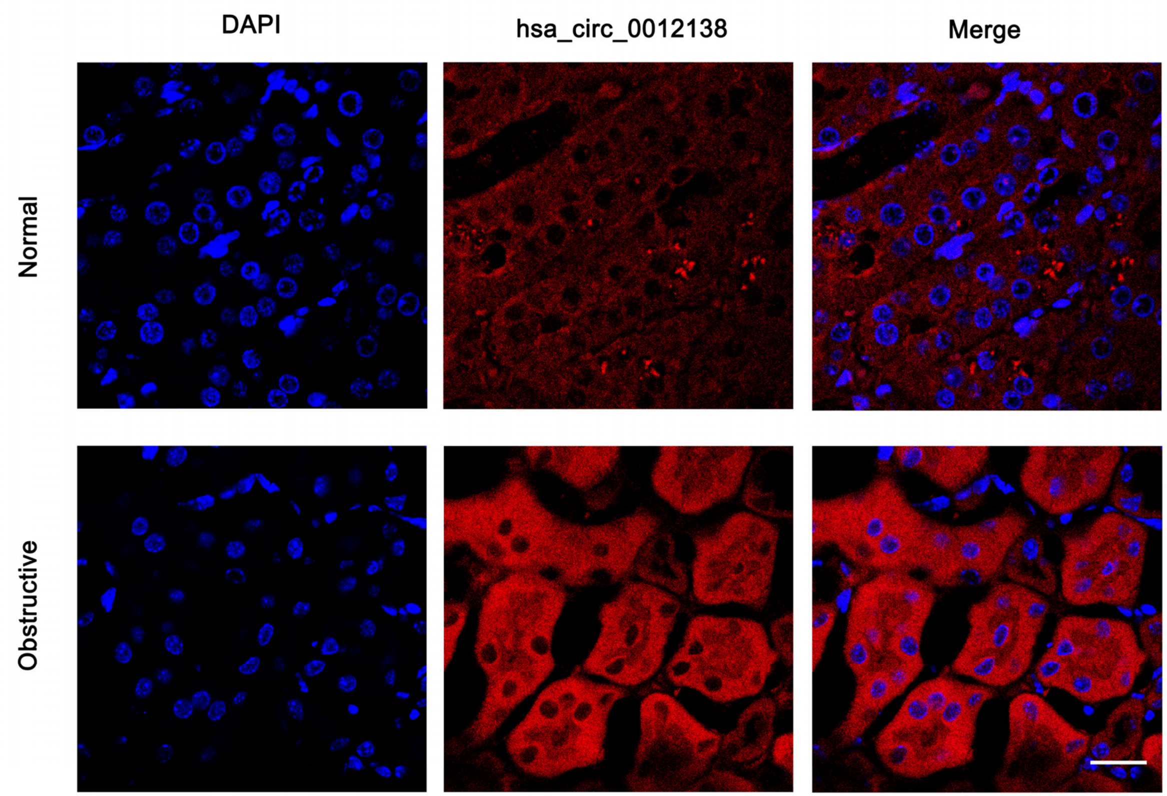

Figure S7. RNA FISH analysis of has_circ_0012138 localization in human renal tissues. (A) Normal kidney. (B) Obstructive nephropathy kidney. Scale bar, 50μm.

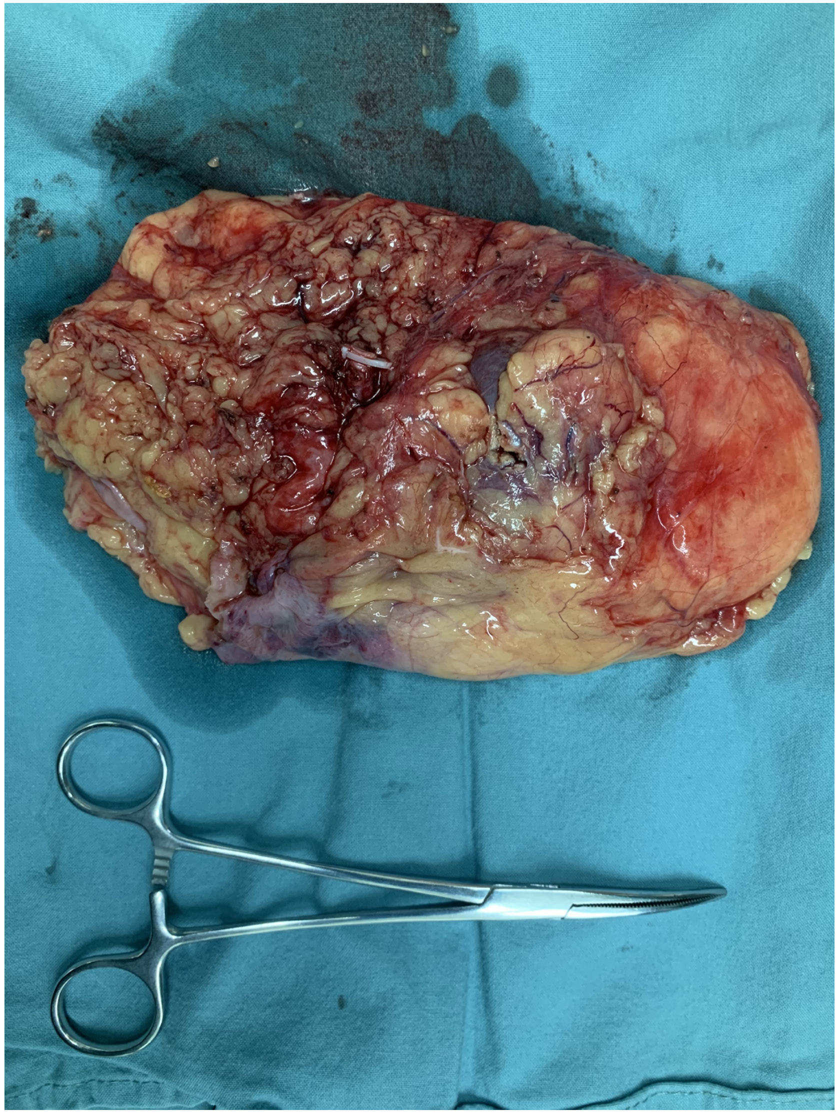

Figure S8. Gross specimen of the resected hydronephrotic kidney.

Table S1. mmu_circ_37492 vs hsa_circ_0012138

organism	position	strand	circRNA ID	genomic length	spliced length	best transcript	gene symbol
hsa	chr1:44713610-44804994	-	hsa_circ_0012138	91384	720	NM_024066	ERI3
mmu	chr4:117564620-117649406	+	mmu_circRNA_37492	84786	720	NM_080469	Eri3

Table S2. General patient characteristics

	Control				Moderate				Severe			
	1	2	3	4	1	2	3	4	1	2	3	4
Age (year)	44	47	57	62	47	51	60	58	66	62	54	63
Gender	male	male	male	male	female	male	female	male	male	female	male	female
Side	right	left	left	right	right	left	left	right	right	right	right	left
Weight (kg)	73	72	70	66	53	64	50	74	78	64	75	70
Height (m)	1.75	1.7	1.68	1.67	1.62	1.68	1.6	1.72	1.68	1.6	171	1.75
*Time to symptoms (week)	/	/	/	/	2	1	2	2	4	3	4	12
#Unilateral residual GFR (mL/min)	/	/	/	/	14.6	14.3	11.7	13.4	9.38	7.36	9.36	6.64
Cortical thickness (mm)	7.2	7.3	6.6	6.4	3.2	4.1	4.4	3.1	2.2	2.5	1.5	1
*symptoms include abdominal mass, distension or pain												
Group A: Time to symptoms<2week, Unilateral residual GFR>10 mL/min, Cortical thickness>3m; Group B:Time to symptoms>2week, Unilateral residual GFR<10 mL/min, Cortical thickness<3mm												

Table S3. Upregulated hsa_miRs-target genes

ID									
ZNF845	SDCBP	DYNLT3	KLHL13	C15orf63	GSTP1	TBC1D9	CNIH2	STAT1	GBP3
C14orf169	PSMD7	BCL3	ALDH18A1	ORMDL2	IL13RA1	CNIH	IRF2BP2	DPY30	GLIPR2
HNRNPU	TRAM1	FBXO8	PFN1P2	ANXA5	CIB2	FAM60A	MBD2	PDIA3	F12
LRRC42	BLZF1	YBX1	FAM115A	NMI	TMEM238	RRAGC	C20orf29	FBXO3	LACTB2
HDAC2	CMTM6	KIAA0020	ARL2BP	STRBP	CDC26	NKX2-8	HOMER3	TMSB10	TSPYL5
ZBTB3	UBC	BCL10	MGAT2	DCDC2	FUCA1	NAA50	LIPC	CDH3	ERMP1
IFNAR1	SEC63	METAP2	XBP1	GBP6	MED4	MTF1	CIT	ACBD7	ATP9A
WDR3	TMEM9B	CTR9	ZNF264	TMEM59	MRPL44	ASB16	HIF1A	LARP7	CASP1
LLPH	PTMA	CHERP	KLF11	SLC27A1	RSPH1	KCTD1	HS3ST1	LAP3	BIRC3
PAPSS1	MANF	DPF2	FRG1	B4GALT5	IQGAP1	PLEKHF1	GLOD4	NUAK1	CDH24
MED10	PPP2R5E	UBXN8	RNF149	ARF4	MAN2B1	CAPZA1	BAMBI	NFATC1	ANAPC13
RAD23B	TMED7	MGAT4A	C9orf25	PTRH2	OMA1	LCE1A	DNAJC10	TIMP2	PROM1
TCF20	STARD3NL	GNAI3	PARP1	ZCCHC7	PLRG1	KCNS3	ARL6IP1	OR5L2	HSD17B11
ZBTB45	TCEA1	C19orf68	ICK	RBM3	GPX7	HOXA3	ZMIZ1	OCLN	GALNT3
EIF3C	TANK	LRRC8A	XPOT	MAP2K4	CANX	ARSJ	LTF	ANXA3	CMTM3
PAM	TMEM43	CLDN3	LTA4H	C12orf39	PDCD4	KHSRP	MRAS	KIAA0101	UCHL1
TMX4	HN1	AGFG1	NFE2L2	MECP2	UBE2Z	QPCT	FAM117B	CYP26B1	LRRC19
ACTR3	TCEAL8	SNAPC2	PNRC2	ARL8B	S100A13	SOX9	ATP6V1A	UBE2C	C8orf4
HSP90B1	GGPS1	LAMP1	COPS2	LAPTM4B	FAM104B	ZFPM1	GDF15	CYP2B6	MORF4L1
TMX1	ITFG3	IFIT2	RPS13	STRAP	POLR2J2	PGRMC2	C9orf62	SOX17	TRIM2
SRFBP1	SNX10	ATAD3C	NUP35	RPLP2	SPSB2	ALDH1A1	DLD	ITGB1	EMP1
BTF3	YWHAB	NCK1	SC5DL	GLUL	APOO	TNFAIP8	SPON2	ZNF157	MVP
WDR70	FANCF	HNRNPF	TMEM50A	WFDC2	LDOC1L	ID4	CENPF	S100A1	PIK3R3
ANXA4	EFTUD1	COMMD8	RHOA	LZTFL1	TES	CAST	CCDC72	LOC100507218	TUBB3
QTRTD1	NME7	MYL6B	SYCE3	PNMA1	LAMC1	SATB1	KGFLP1	LOC100130557	SAMD9L
NBN	ARFGAP3	TMEM33	SCAF8	ZMPSTE24	COL4A1	ERP27	ENPP5	DIRC1	PHLDA2
FOXC2	GPR156	FAM45A	RAB3IP	PTPLB	C20orf30	C6orf72	ARHGAP24	PHOX2A	HIST1H4E
BEST4	C20orf141	YWHAH	CLUAP1	GLB1	SOCS1	USP1	HTATIP2	KRTAP5-8	MLEC
RAB18	ELP4	SUMO2	44454	S100A10	F11R	HMGB2	DUX4	C1QBP	TM4SF4
VPS35	KDM6A	C12orf5	VPS26A	TBK1	TMEM125	RAB21	UGCG	CST6	SNCB
PPIC	SNX2	KDM3B	PTP4A1	MDK	EIF4E	DOCK1	TM4SF18	PCP4	LOC92249
ABI1	PAIP1	TXNDC9	SWAP70	PCNA	EXOC6	FAM129B	RAI2	C11orf58	C3orf54
ARHGAP1	MESDC2	RPUSD2	CEBPZ	COL4A2	CLN5	BCLAF1	SLC9A3R2	RAP1A	MAB21L2
ZNF644	VWA1	CYB5R3	AP3S1	PTPRK	KCNJ16	MSH6	ZNF503	TTLL3	ZNF541
JUND	STOM	PPDPF	UBE2A	CCT2	TLR3	RAB3GAP2	RPS26	FBLL1	IFI16
FAM199X	RARS	SYNCRIP	CKAP2	CALR	RASSF10	CHMP1B	FAIM	PTTG1	ORAI2
LOC100652765	LZTS2	MEAF6	SFR1	STMN1	FGF7	BIVM	C1GALT1C1	HIBADH	TRIM6
ALG13	RAB11FIP1	PHOSPHO2	SKP1	GAR1	LAD1	PLSCR1	HIST1H2AC	ZMAT2	SLC22A2
LOC100129292	SCARNA17	RELB	CSDE1	TMEM201	TTC39A	ZFP90	CAMK2N1	C9orf125	LOC100506758
CIR1	TDRD7	GOLGA5	ANXA7	F8A1	PLBD1	EDN1	SCHIP1	S100A2	RNF144B
TMED2	NF2	ZNF14	TAF6	TRIP12	BBC3	C15orf48	EDN2	CDH1	PRSS36
ADAM10	PHAX	H2AFV	COX16	RNF130	TARS	ZFP36L2	ACSL1	IL27	PERP
MPZL1	DKC1	GPATCH4	KATNA1	SOX4	MYC	C6orf48	CRYAB	LTB	FOLR1
FNDC3B	C11orf10	SHISA5	TWF1	OSBPL10	SGMS1	SCPEP1	C4orf48	FOXB1	TMPRSS4
C14orf166	TSN	NUCB2	SMARCE1	FBXO34	C9orf167	IFI30	SLC44A1	MARCKS	SLC16A5
UBE2D1	TLR1	GTF2B	SPPL3	SEC61B	CALM2	FAM84B	ITM2B	EPCAM	VNN1
LRFN3	TMEM184C	C5orf41	COPB2	NSRP1	LOC100652733	MIF	TMEM132A	HIST1H4B	HP
DNTTIP2	HNRNPH2	MVK	WBP4	SCRT2	PAOX	SPCS2	SNAP23	SCP2	NKAIN4
MYL12A	SEC62	44446	LMCD1	VIM	ARL6IP5	CNN3	NPTN	ANXA1	CPVL
TGIF1	CD59	NMT2	VAT1	SNX6	TXNDC5	PNRC1	CRYZ	ABR	GCNT3
DIRC2	CASP4	SHOC2	CASP5	GALNT7	TK1	CASP7	VCAM1	SPP1	MT2A
PDIA4	NDNL2	KTN1	LIPA	LEPROTL1	NDUFAF3	AMOTL2	SIAE	SYF2	XIST
HNRNPA3	MLF1	TMEM106C	PRMT6	ATP6V1G1	C12orf75	TMEM54	SPCS3	CDT1	CFI
PPP3CA	DYRK3	KRT8	UTP3	CX3CL1	HMGB3	C15orf39	SUSD1	BCHE	SLC22A18AS
BRCC3	CDC42	RBM7	CCDC59	MYOF	ROMO1	NTN4	SAR1B	NME5	ASAH1
MAFA	ATP2C1	IFIT5	LOC100134167	DEPTOR	PIGK	FUT8	RPN1	CD24	CXCL1
GRN	C6orf165	HMGN4	RAB36	SEL1L	HEXIM1	HMGN5	TRIB3	S100A6	SPARCL1
TMCO3	MUTED	SMS	ANO6	PLK1S1	DCP1B	CASP8	NUAK2	WBP5	GPM6A
NLN	TMEM48	CPLX2	EAPP	VMP1	TNFRSF12A	CXorf36	RELL1	SOCS3	SAMD10
DTD1	LMAN1	PLK2	TNFSF10	CD63	TCEAL1	E2F3	DDX58	SNHG5	NNMT
H2AFX	ARPC5	SLC15A4	GLIS3	EBPL	ERGIC1	IL4I1	C5orf58	HIST1H4L	MX1
DENR	DDX27	RPL38	MMP7	OLA1	SERINC1	ETFDH	SOD2	AMBP	CDH6
HPS6	EIF4A3	LINC00094	NPEPPS	HSPB9	FLRT3	SP3	SEL1L3	SERPINE2	SLC39A8
FGB	PHF3	PRPF18	ADAM9	FAM101B	ZNF713	NPC2	VSIG10L	HIST1H4C	ISG20

Table S4. Primers for RT-qPCR

circRNA_37492	5'-CATGTATGTCCAGCCTGTAGTCCAC-3'(forward)
	5'-CTTCCTTCGCCATCCACTCATCG-3' (reverse)
miR-7682-3p	5'-CGCCTGTGGGTTGGGTT-3' (forward)
	5'-AGTGCAGGGTCCGAGGTATT-3' (reverse)
Fgb	5'-ATCTATGGCTGCTGCTGCTATTGTG-3' (forward)
	5'-CAGGTCTTAGGCTAGGAGGCTCTTC-3' (reverse)
hsa_circ_0012138	ACATGAACAAGGGCCTCAGC (forward)
	ATCGAGCTGCTCCAGTCTGT (reverse)
miR-651-5p	5'- GCGCGTTTAGGATAAGCTTGA-3' (forward)
	5'- AGTGCAGGGTCCGAGGTATT-3' (reverse)
FGB	5'-ACCTTCGTGTGCTTCGTTCAATCC-3' (forward)
	5'-TGCCAGACACCACAGGAATATTGC-3'(reverse)
bβ-actin	5'-GCACAGGGTGCTCCTCAG-3' (forward)
	5'-CTAGGCACCAGGGTGTGATG-3' (reverse)
U6	5'-GGCCTATTTCCCATGATTCC-3'(forward)
	5'-ATTTGCGTGTCATCCTTGC-3'(reverse)

Table S5. Sequence of circRNA_37492

>mmu_circRNA_37492
TTTTAGATGCCTCTGGATGTTCAATGCTAGCGCCGTTACAGACGGGAGCGGCTCGGTTCT
CTTCATATTTACTTTCAAGAGCAAGAAAAGTGCTGGGCTCCCACTTGTTATCTCCCTGTG
GCGTTCCGGAGCTCTGTTCCATATCCACCAGAAAGCTGGCGGCCCACGGCTTTGGCGCGG
CAATGGCGGCAATGGTGCCCTTCCCTCCCCAGAGGTATCACTACTTTTTAGTGCTGGACT
TTGAGGCCACATGCGACAAGCCACAGATCCACCCTCAGGAAATCATTGAGTTCCCCATCC
TGAAGTTAAATGGCCGGACCATGGAAATCGAGTCTACCTTTCACATGTATGTCCAGCCTG
TAGTCCACCCACAGCTTACCCCCTTCTGTACAGAGCTCACCGGGATCATTCAAGCCATGG
TGGATGGCCAGCCAAGCCTGCAGCAAGTGCTGGAGAGGGTCGATGAGTGGATGGCGAAGG
AAGGCCTCTTAGATCCAAACGTCAAGTCAATCTTTGTCACCTGCGGAGACTGGGACCTGA
AAGTCATGCTTCCAGGACAGTGCCATTATCTAGGCTTGCCAGTGGCGGATTACTTCAAGC
AGTGGATTAATCTAAAAAAGGCTTACAGCTTCGCCATGGGCTGCTGGCCCAAGAATGGAC
TACTAGACATGAACAAGGGTCTCAGCCTGCAGCACATAGGCCGGCCCCACAGCGGCATTG
