# Supplementary figures and images for: The mmu_circRNA_37492/hsa_circ_0012138 function as potential ceRNA to attenuate obstructive renal fibrosis
Source: Cell Death Dis. 2022 Mar 4;13(3):207. doi: 10.1038/s41419-022-04612-3 (PMC8897503; doi:10.1038/s41419-022-04612-3)

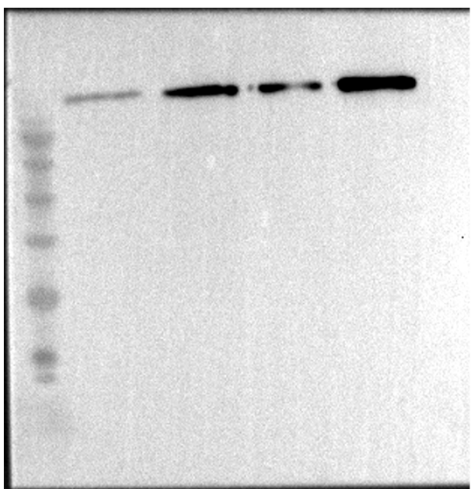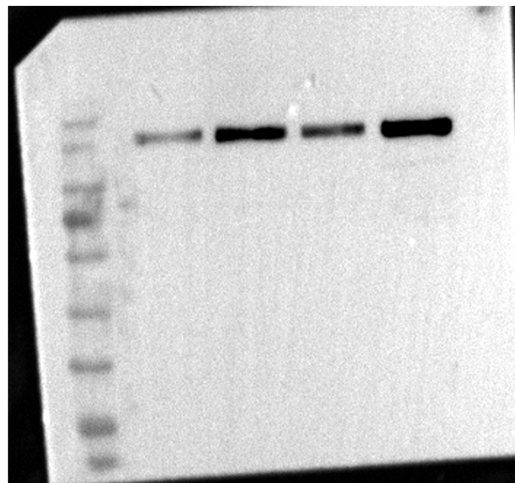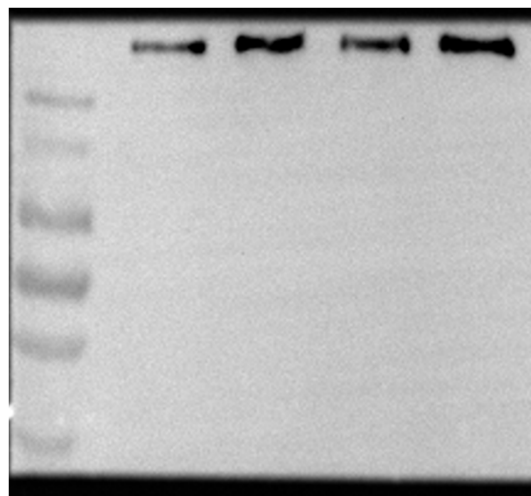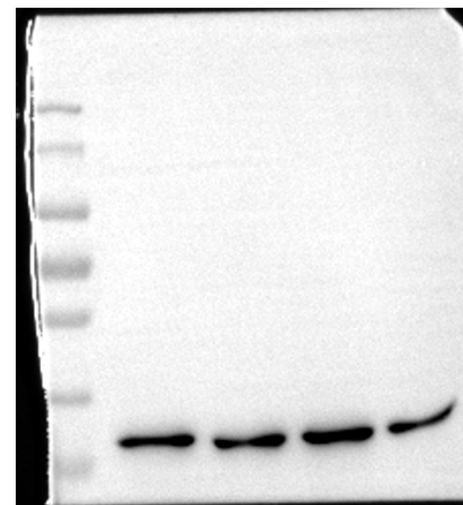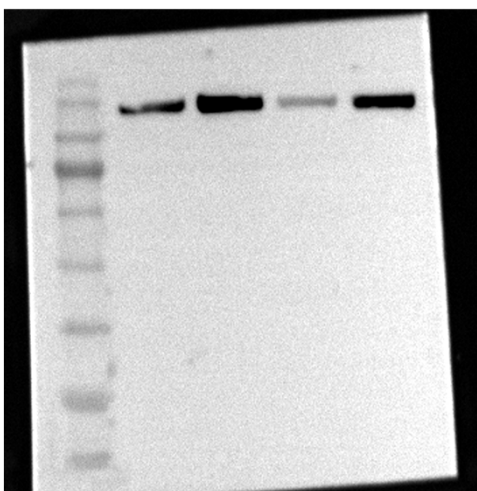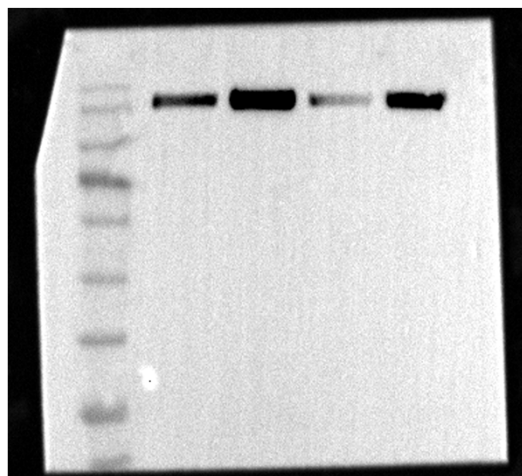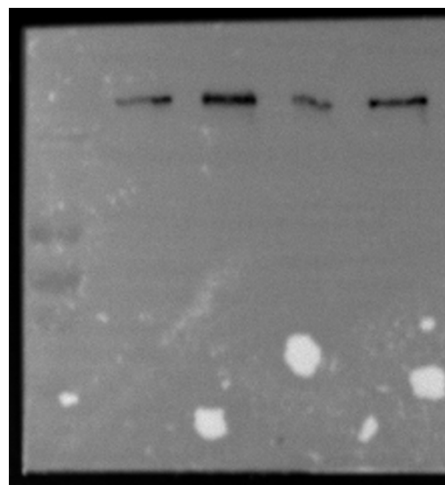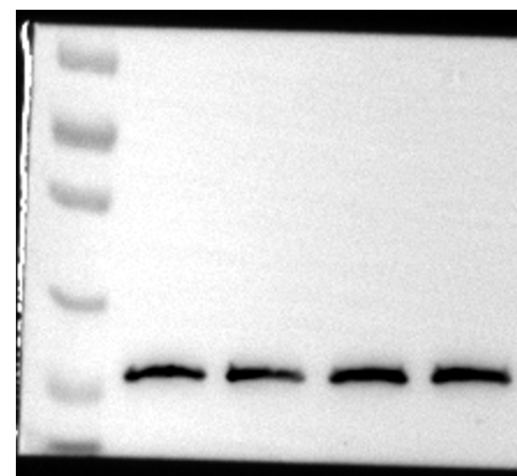

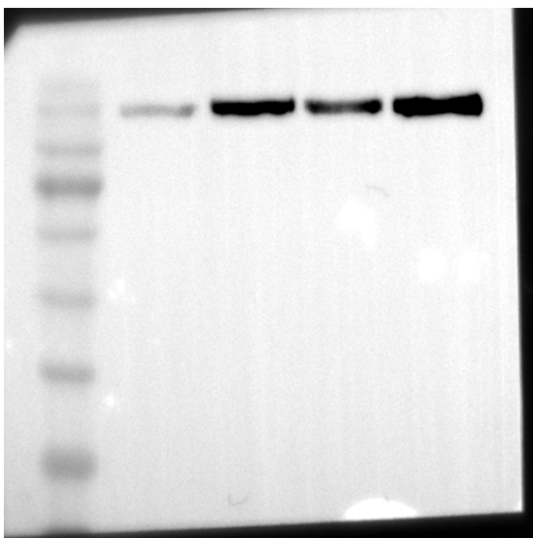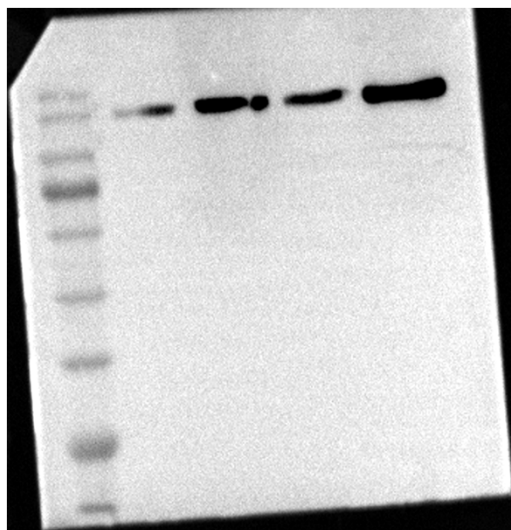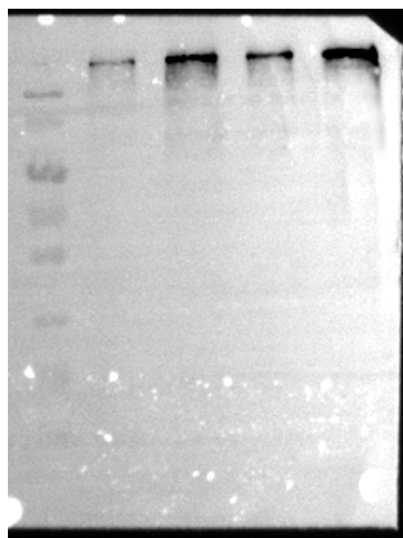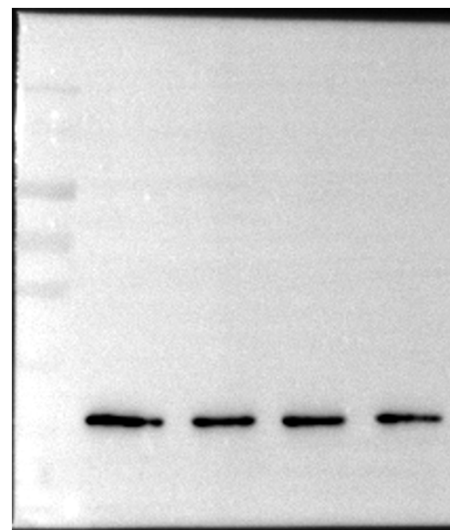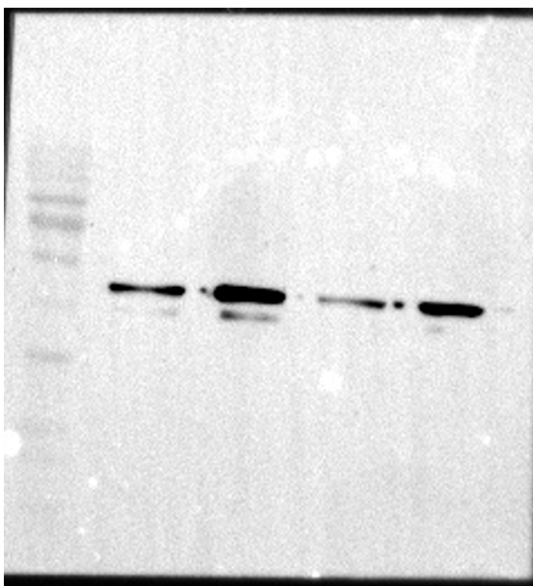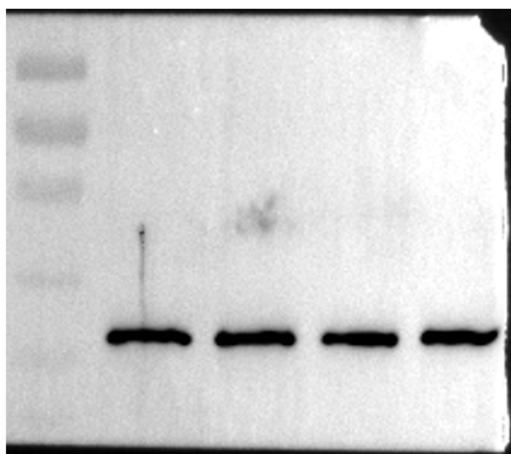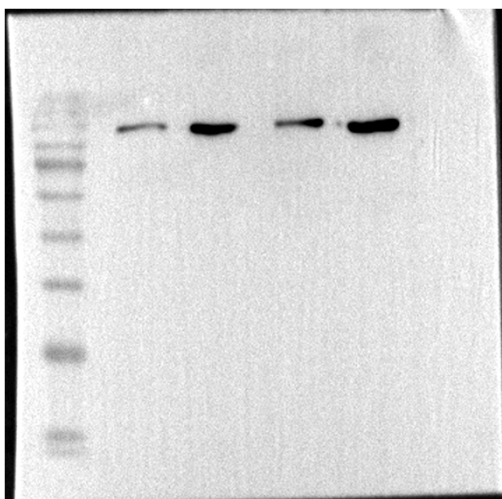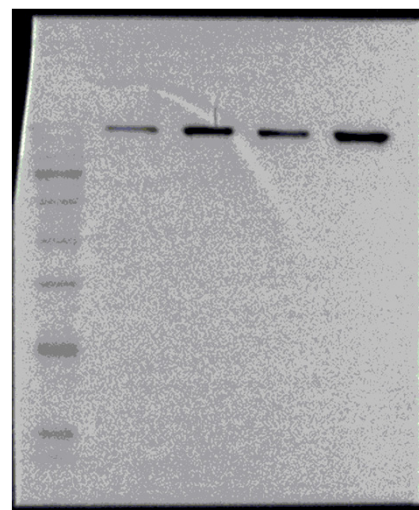

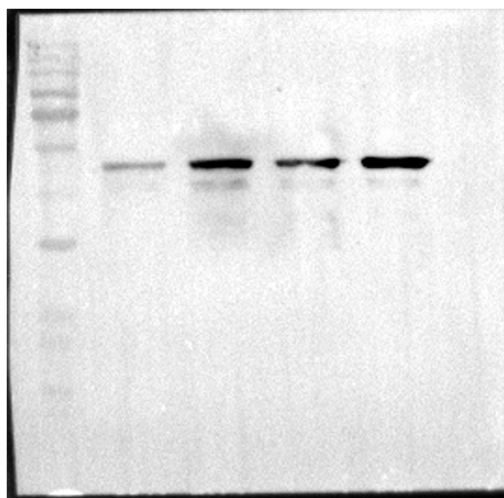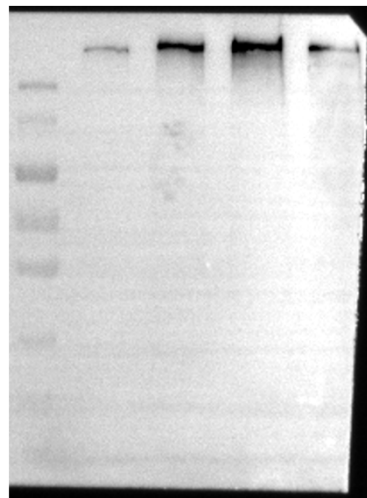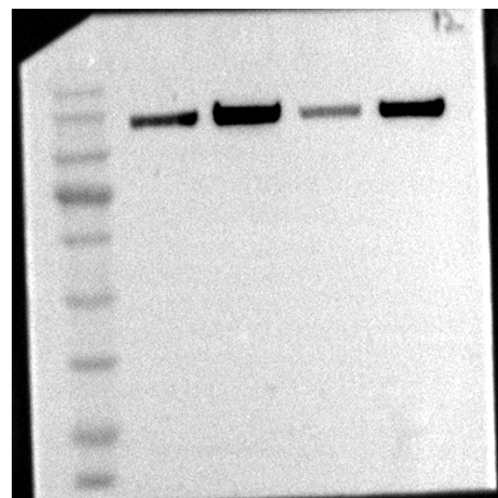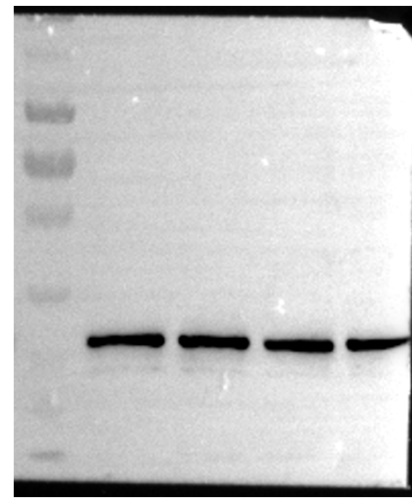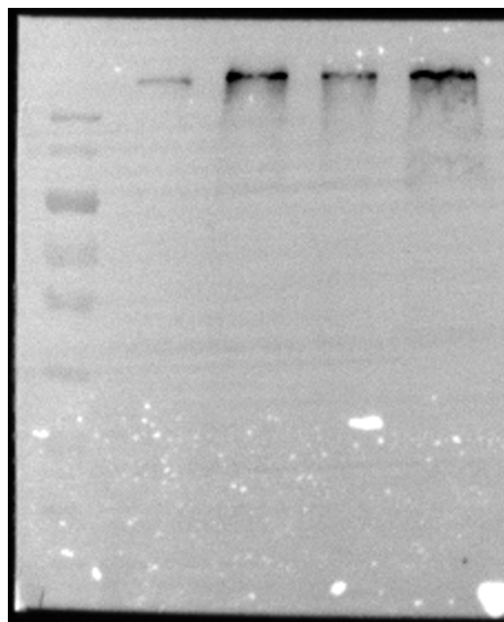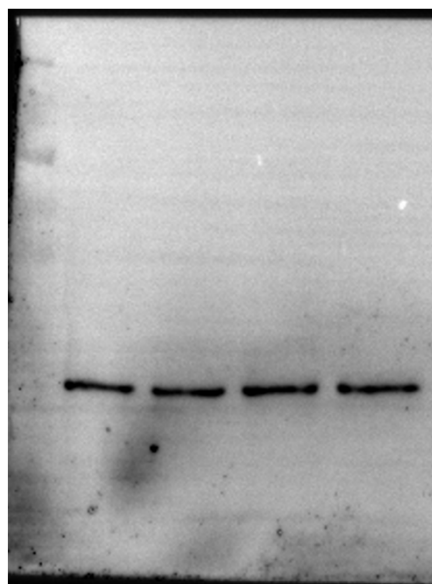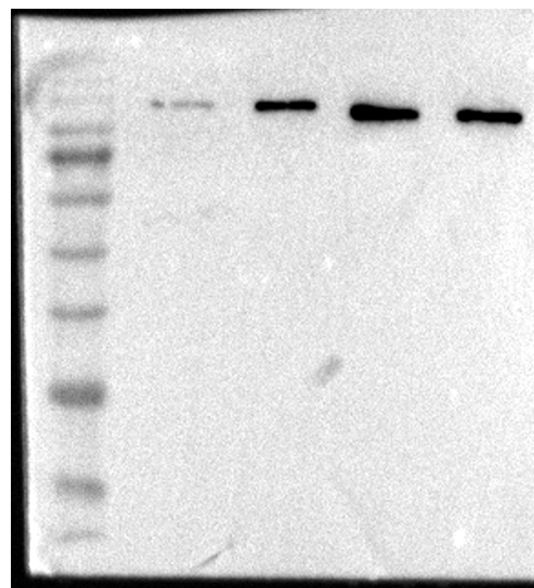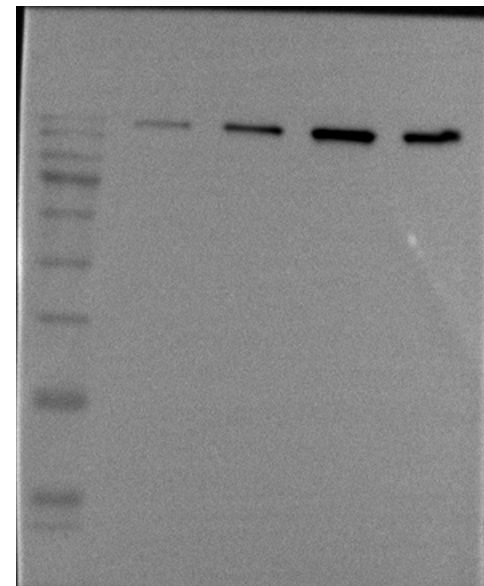

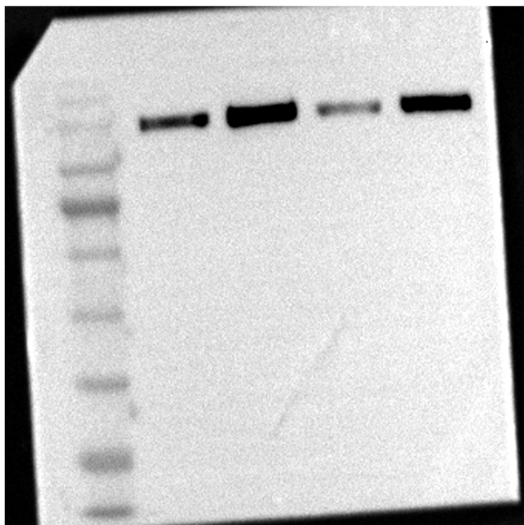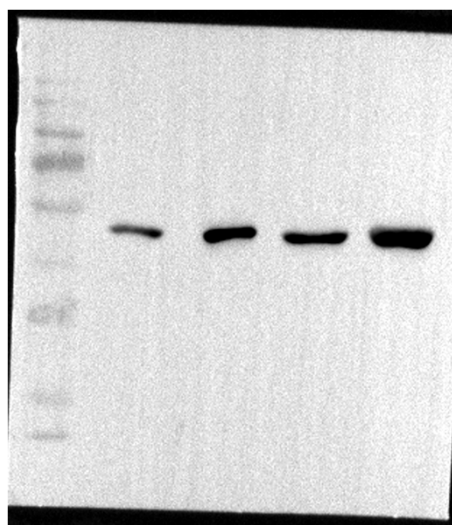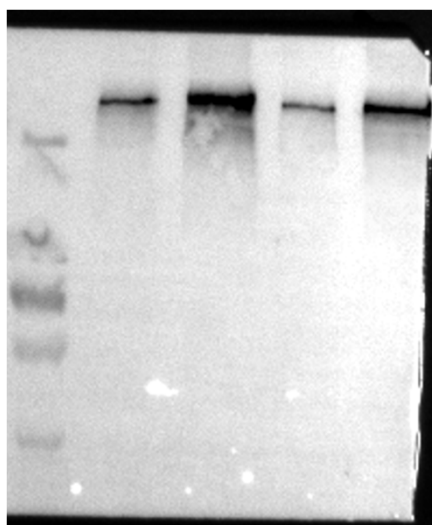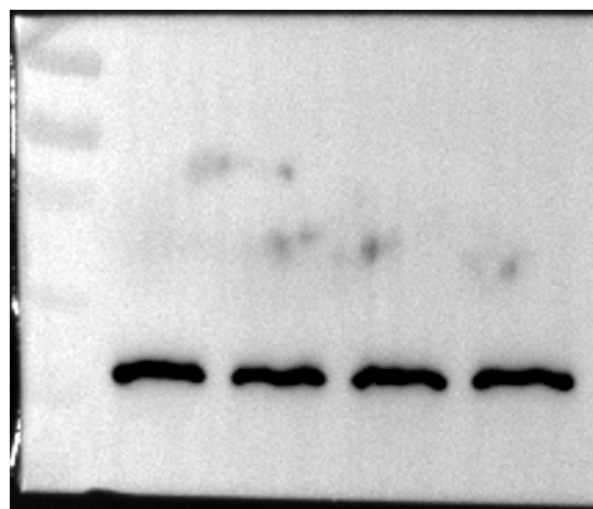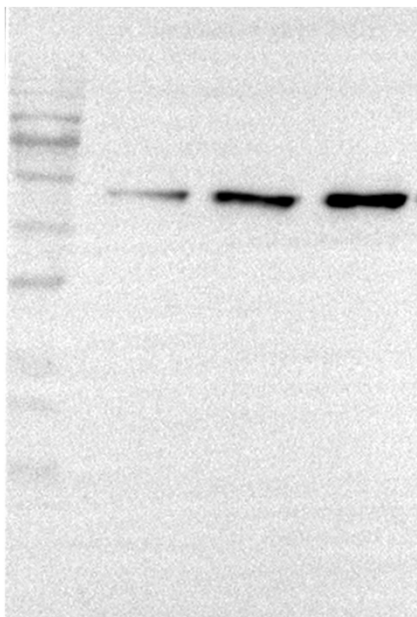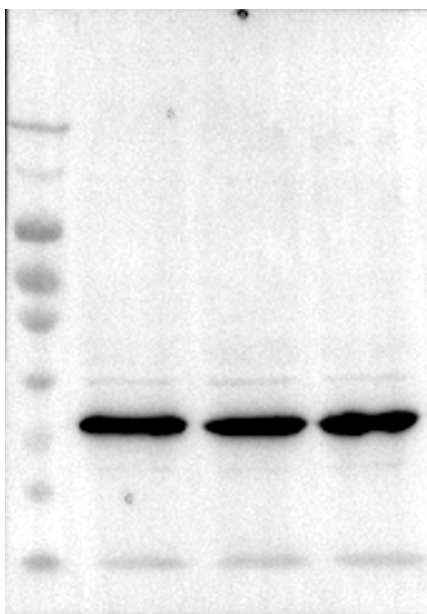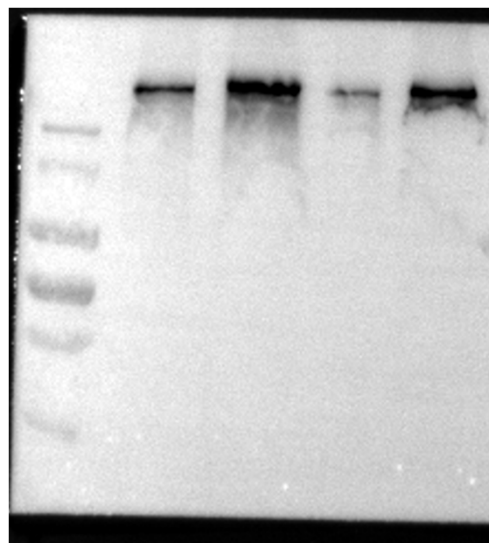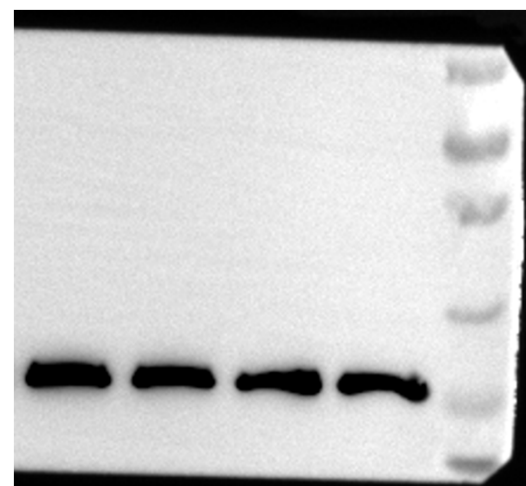

Supplement: Supplementary file 2 — Original Data [file 41419_2022_4612_MOESM2_ESM.pdf]
